# Improved gene delivery to K-562 leukemia cells by lipoic acid modified block copolymer micelles

**DOI:** 10.1186/s12951-021-00801-y

**Published:** 2021-03-06

**Authors:** Friederike Richter, Prosper Mapfumo, Liam Martin, Jana I. Solomun, Franziska Hausig, Jochen J. Frietsch, Thomas Ernst, Stephanie Hoeppener, Johannes C. Brendel, Anja Traeger

**Affiliations:** 1 Laboratory of Organic and Macromolecular Chemistry (IOMC), Friedrich Schiller University Jena, Humboldtstrasse 10, 07743 Jena, Germany; 2 Klinik für Innere Medizin II, Abteilung Hämatologie und Internistische Onkologie, Universitätsklinikum Jena, Am Klinikum 1, 07747 Jena, Germany; 3 Jena Center for Soft Matter (JCSM), Friedrich Schiller University Jena, Philosophenweg 7, 07743 Jena, Germany

**Keywords:** Gene delivery, Cationic polymer, Lipoic acid, Micelle, K-562 cells, Transfection

## Abstract

Although there has been substantial progress in the research field of gene delivery, there are some challenges remaining, e.g. there are still cell types such as primary cells and suspension cells (immune cells) known to be difficult to transfect. Cationic polymers have gained increasing attention due to their ability to bind, condense and mask genetic material, being amenable to scale up and highly variable in their composition. In addition, they can be combined with further monomers exhibiting desired biological and chemical properties, such as antioxidative, pH- and redox-responsive or biocompatible features. By introduction of hydrophobic monomers, in particular as block copolymers, cationic micelles can be formed possessing an improved chance of transfection in otherwise challenging cells. In this study, the antioxidant biomolecule lipoic acid, which can also be used as crosslinker, was incorporated into the hydrophobic block of a diblock copolymer, poly{[2-(dimethylamino)ethyl methacrylate]_101_-*b*-[*n*-(butyl methacrylate)_124_-*co*-(lipoic acid methacrylate)_22_]} (P(DMAEMA_101_-*b*-[*n*BMA_124_-*co*-LAMA_22_])), synthesized by RAFT polymerization and assembled into micelles (LAMA-mic). These micelles were investigated regarding their pDNA binding, cytotoxicity mechanisms and transfection efficiency in K-562 and HEK293T cells, the former representing a difficult to transfect, suspension leukemia cell line. The LAMA-mic exhibited low cytotoxicity at applied concentrations but demonstrated superior transfection efficiency in HEK293T and especially K-562 cells. In-depth studies on the transfection mechanism revealed that transfection efficiency in K-562 cells does not depend on the specific oncogenic fusion gene *BCR-ABL* alone. It is independent of the cellular uptake of polymer-pDNA complexes but correlates with the endosomal escape of the LAMA-mic. A comparison of the transfection efficiency of the LAMA-mic with structurally comparable micelles without lipoic acid showed that lipoic acid is not solely responsible for the superior transfection efficiency of the LAMA-mic. More likely, a synergistic effect of the antioxidative lipoic acid and the micellar architecture was identified. Therefore, the incorporation of lipoic acid into the core of hydrophobic-cationic micelles represents a promising tailor-made transfer strategy, which can potentially be beneficial for other difficult to transfect cell types.
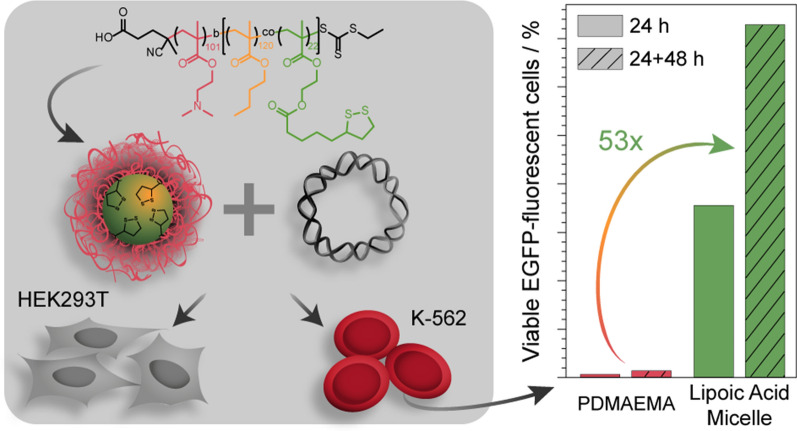

## Introduction

Non-viral gene therapy has become an important research field, with the aim of finding new methods for the treatment of diseases like cancer or genetic disorders or for the development of vaccines [1, 2]. Cationic polymers are of interest due to their ability to form complexes with the genetic material (polyplexes) through electrostatic interaction, thereby protecting it from degradation, facilitating its cellular uptake, enabling escape from the endolysosomal pathway, and finally releasing it inside the cytosol [3]. Furthermore, polymers are easy and cost-effective to produce on a large scale, show low immunogenicity and can transport high molecular weight genetic materials, such as plasmid DNA (pDNA) [4, 5].

Despite remarkable progress, there are still many challenges encountered for these applications, e.g. not all cell types can be genetically modified (transfected) easily, including primary cells and suspension cells (immune cells) [6–8]. To date, viral infection, electroporation or nucleofection have been successfully applied for these cells. However, this is not suitable for all applications [4, 9, 10]. Alternatively, cationic polymers can be applied to transfect cells. The ability to genetically modify suspension cells is of great interest, not only for vaccinations, inflammation-related diseases or cancer in general, but also for leukemia patients where the immune cells are directly affected. For example, chronic myeloid leukemia (CML) can still only be clinically healed but not cured, with 40 % of patients free of treatment after therapy [11–13]. Polymeric nanocarriers could be a promising strategy to interact more efficiently with immune cells and enhance the intracellular concentration of active agents to counteract mechanisms such as drug resistance or poor response to treatment [14, 15]. In this regard, the group of E. Wagner could already show successful transfection of pDNA with poly(ethylene imine) (PEI) only in combination with transferrin [16–18]. Another promising strategy was demonstrated by H. Uludağ and coworkers who statistically incorporated hydrophobic moieties into polymeric nanocarriers that were beneficial for the transfection of siRNA and subsequent knockdown of the BCR-ABL fusion protein in the CML cell line K-562 [19–21].

One key advantage of polymeric gene carriers is their versatility in composition, allowing the introduction of different additional features such as temperature-, pH- or redox-responsiveness, thereby generating nanocarriers tailored for a specific site of action [5, 22]. The advancement of living or controlled polymerization methods resulted in preparation of polymers with various compositions and complex architectures such as, gradient, block, star and comb copolymers [23]. Among reversible deactivation radical polymerization (RDRP) methods, reversible addition fragmentation chain transfer (RAFT) polymerization, a process based on an equilibrium between active and dormant chains achieved by a degenerative transfer system, is promising due to the relative insensitivity towards functional groups of the monomers and can therefore be applied for a wide range of suitable monomers. However, disulfide bonds are a potential exception since they are susceptible to radicals [23, 24]. Additionally, RAFT polymerization is compatible with a wide range of unprotected monomer functionalities, such as quaternary amino, carboxylic acid, epoxy, hydroxyl (e.g. in hydroxyethyl methacrylate (HEMA)) and tertiary amino (e.g. in 2-dimethylaminoethyl methacrylate (DMAEMA)) groups. This makes the production of polymers with the desired functionalities easier compared to, e.g. the post-modification strategy of polymers to obtain defined polymer architectures [25].

Polymeric micelles with various compositions have already been investigated for gene delivery [26–29]. Due to the amphiphilic nature of the block copolymers, microphase separation occurs during self-assembly, resulting in micellar formation of diverse morphologies [30]. A commonly applied method for polymer assemblies is dispersion of polymers, which involves solvent evaporation, salting-out, dialysis, supercritical fluid technology and nanoprecipitation [31].

In general, polymeric micelles for gene delivery contain a hydrophilic cationic segment, responsible for condensing the genetic material and protecting it from degradation, e.g. PDMAEMA with a p*K*_a_ (≈ 7.5) within a physiologically relevant pH range [32, 33]. Besides, micelles also contain a hydrophobic segment responsible for its stability and potentially contributing to efficient transfection as they can facilitate interaction with the lipophilic cell membranes promoting cellular uptake and release from the endosomes [34–36]. Among others, *n*-butyl methacrylate (*n*BMA) has been shown to form a stable hydrophobic core and, as such, been included for hydrophobic modifications of polymers and formation of micelles [36–38].

To stabilize the micelle architecture, crosslinkers such as disulfide linkage have been applied. Disulfide linkages are stable under oxidizing conditions and break down under reductive conditions, which are found inside cells [39]. A stable micelle can therefore be prepared by incorporating sulfur containing moieties, e.g. the antioxidant biomolecule lipoic acid, which is an essential fatty acid, a potential therapeutic [40, 41], and capable of forming disulfide linkages in the micellar core [42]. For drug delivery systems, Zhong and coworkers demonstrated that different lipoic acid conjugated materials possessed superior stability in the extracellular environment and underwent rapid de-crosslinking and disassembly under reductive conditions [39, 43–45]. Moreover, lipoic acid was beneficial for transfection of siRNA or pDNA in different adherent cell lines when incorporated into nanogels [46], as hydrophobic modification of linear poly(ethylene imine) (LPEI) [47], or as amphiphiles [48, 49]. However, the contribution of lipoic acid within micelles to gene transfer into immune cells is not known.

In the present study, lipoic acid derived micelles were investigated as a potent nanocarrier for genetic material into the CML cell line K-562. The micelles were formulated from a block copolymer synthesized via RAFT polymerization, the first block comprising PDMAEMA and the second lipoic acid methacrylate (LAMA) and *n*BMA. Whereas PDMAEMA forms the shell and contributes to pDNA binding and endosomal escape, *n*BMA forms the micellar core into which lipoic acid-containing monomers were introduced to establish antioxidant and crosslinking potential. Following physicochemical characterization, the pDNA binding properties, cytotoxicity, and transfection efficiency (expression of enhanced green fluorescent protein (EGFP)) in the erythroleukemic suspension cell line K-562 were investigated. The results were compared to the human embryonic kidney cell line HEK293T. The contribution of lipoic acid to the superior transfection efficiency in K-562 cells was investigated in detail by further testing of other leukemia cell lines as well as pDNA uptake and endosomal escape properties. Additionally, the influence of structure and composition was investigated by comparing the potential of the LAMA-micelles with lipoic acid-free precursor micelles.

## Main methods

Materials, instruments, further methods and calculations can be found in the Additional file 1.

### Synthesis and characterization, general procedure

(4-cyano pentanoic acid)yl ethyl trithiocarbonate (CPAETC) (24.9 mg, 9.47 × 10^− 5^ moles), 2-(dimethylamino)ethyl methacrylate (DMAEMA) (2.25 g, 1.43 × 10^− 2^ moles), 1,4-dioxane (3.0 g), a 1 wt.% solution of 4,4′-azobis(4-cyanovaleric acid) (ACVA) in 1,4-dioxane (318,4 mg, 3,18 mg ACVA, 1.1 × 10^− 5^ moles) and 1,3,5-trioxane (external NMR standard, 25 mg) were introduced to a 8 mL microwave vial equipped with a magnetic stirring bar. The vial was sealed, and the solution deoxygenated by bubbling argon through it for 10 min. The vial was placed in an oil bath at 70 °C and allowed to stir for 7 h. The polymer was precipitated three times from tetrahydrofuran (THF) into cold hexane and dried under reduced pressure to give a yellow solid. A portion of the precursor, macro-chain transfer agent (macro-CTA) of poly[2-(dimethylamino)ethyl methacrylate] (PDMAEMA) (347.0 mg, 2.29 × 10^− 5^ moles), butyl methacrylate (*n*BMA) (557.0 mg, 3.92 × 10^− 3^ moles), lipoic acid methacrylate (LAMA) (220.0 mg, 6.88 × 10^− 4^ moles), THF (4.1 g), a 0.5 wt.% solution of ACVA in THF (250 mg, 4.46 × 10^− 6^ moles) and 1,3,5-trioxane (external NMR standard, 20.0 mg) were introduced to a 8 mL microwave vial equipped with a magnetic stirring bar. The vial was sealed, and the solution deoxygenated by bubbling argon through it for 10 min. The vial was placed in an oil bath at 70 °C and allowed to stir for 7 h. The polymer was precipitated three times from THF into cold hexane and dried under reduced pressure to give a yellow solid. For further experimental details of all polymerizations refer to Additional file 1.

### Assembly procedure for micelle formation

*Typical assembly procedure.* 30 mg of polymer was dissolved in THF (3 mL) and added to a 20 mL vial. Water (6 mL) was added to this solution through a syringe using a syringe pump (rate: 0.2 mL min^− 1^). Afterwards, (A) the polymer solution was added to a dialysis bag (Standard RC Tubing MWCO: 6–8 kDa) and dialyzed against water over three days, changing the bulk water twice a day or, (B), the polymer solution was left for two days at room temperature until THF was completely evaporated. The final concentration was determined by measuring the mass difference of three freeze dried samples of known volume. The micelles were characterized regarding their critical micelle concentration (CMC), hydrodynamic diameter and morphology using Nile Red as a fluorescent probe, dynamic light scattering (DLS) and cryo transmission electron microscopy, respectively. Experimental details for each analysis are provided in Additional file 1.

### Cryo transmission electron microscopy (cryo-TEM)

Cryo-TEM images were acquired with a 200 kV FEI Tecnai G2 20 transmission electron microscope equipped with a 4k × 4k Eagle HS CCD and an Olympus MegaView camera (1379 × 1024 pixels) for overview images. Sample preparation was performed by plunge-freezing the samples with a Vitrobot Mark IV system. 8.5 µL of the aqueous solutions were blotted (blot force − 2; blotting time 1 s) on Quantifoil grids (R2/2, Quantifoil, Jena, Germany) and were vitrified in liquid ethane. The grids were rendered hydrophilic by Ar-plasma cleaning for 30 s (Diener Electronics, Germany). Samples were stored in liquid nitrogen until transfer to the cryo holder (Gatan 626). Transfer to the microscope was performed with a Gatan cryo stage and the temperature was maintained below − 172 °C at all times after vitrification.

### Polyplex preparation

The polyplexes were prepared in HBG buffer (20 mM 4(2hydroxethyl) piperazine-1-ethanesulfonic acid (HEPES) and 5 % (w/v) glucose, pH 7.4). A 30 µg mL^− 1^ solution of pDNA was mixed 1:2 with different quantities of dissolved polymer to give a final pDNA concentration of 15 µg mL^− 1^, with varying N*/P ratios (molar ratio of protonatable nitrogen atoms to phosphates of pDNA, see Additional file 1). Immediately after combination, the mixtures were vortexed for 10 s at maximum speed (3200 rpm) and incubated at room temperature for 15 min to ensure complex formation.

### Ethidium bromide quenching assay (EBA) and heparin release assay (HRA)

The formation of polyplexes with pDNA was identified via quenching of ethidium bromide (EtBr) fluorescence by polymers interacting with pDNA as described before.[50] Briefly, 30 µg mL^−1^ pKMyc pDNA in HBG buffer (pH 7.4) were incubated with EtBr (1 µg mL^−1^) at room temperature for 10 min. Different polymer stock solutions were prepared by dilution with HBG buffer (pH 7.4) to give different N*/P ratios. Subsequently, the pDNA-EtBr solution was mixed 1:2 with the different polymer stock solutions in black 96-well plates (Nunc, Thermo Fisher, Germany) and incubated at 37 °C for 15 min before measuring the fluorescence intensity at *λ*_Ex_ = 525 nm / *λ*_Em_ = 605 nm. A sample containing only pDNA and EtBr was defined as maximum fluorescence (100 %).

For the HRA, heparin was added to the formed polyplex-EtBr mixtures using the dispenser of the microplate reader to obtain the indicated concentrations (Additional file 1: Table S2). After each addition, the plate was shaken, incubated at 37 °C for 10 min and fluorescence intensity was measured.

The percentage of EtBr displaced upon polyplex formation or re-intercalating following pDNA release by heparin was calculated using Eq. (1):

1 $${\text{rFI}}/\% = \frac{{{{\text{F}}_{{\text{Sample}}}}}}{{{{\text{F}}_{{\text{pDNA}}}}}}~ \cdot 100$$

Where rFI is the relative fluorescence intensity and F_Sample_, and F_pDNA_ are the fluorescence intensities of a given sample and the EtBr intercalated into pDNA alone (in the case of the HRA with heparin), respectively. Data are expressed as mean ± SD of three independent determinations.

The heparin concentration needed to release 70 % of pDNA was calculated with OriginPro, Version 2018b (OriginLab Corporation, US) and can be found in the Additional file 1.

### Cell culture

The mouse fibroblast cell line L-929 and the human embryonic kidney cell line HEK293T were obtained from CLS (Germany). They were maintained as recommended by the supplier and cultured in D10 (low glucose Dulbecco’s modified eagle’s medium (DMEM) supplemented with 10 % (v/v) fetal calf serum (FCS), 100 U mL^−1^ penicillin and 100 µg mL^−1^ streptomycin) at 37 °C in a humidified 5 % (v/v) CO_2_ atmosphere. The chronic myeloid leukemia (CML) cell line K-562 was obtained from DSMZ (Germany) and cultured in R10 (Roswell Park Memorial Institute (RPMI) 1640 medium supplemented with 10 % (v/v) FCS, 100 U mL^−1^ penicillin and 100 µg mL^−1^ streptomycin) at 37 °C in a humidified 5 % (v/v) CO_2_ atmosphere. For comparison to other leukemia cell lines and determining the influence of BCR-ABL, the transformed acute myeloid leukemia (AML) cell line M07p210 [51] and the CML blast crisis cell lines LAMA-84 and KCL-22 were analyzed. All cell lines, except for M07p210, were obtained from DSMZ. The cells were cultured as described for the K-562 cells.

For experiments, L-929 and HEK293T cells were seeded at 0.1 or 0.2 × 10^6^ cells mL^− 1^, respectively, in growth medium (D10) containing 10 mM HEPES for stability of the pH value and incubated at 37 °C in a humidified 5 % (v/v) CO_2_ atmosphere for 24 h. One hour prior to transfection, the medium was changed to fresh growth medium with HEPES. Unless stated otherwise, the K-562 and other leukemia cell lines were seeded at 0.3 × 10^6^ cells mL^− 1^ in growth medium (R10) containing 10 mM HEPES and incubated at 37 °C in a humidified 5 % (v/v) CO_2_ atmosphere for about 3 h before transfection.

### Determination of cytotoxicity

For determination of cytotoxicity of the polymers, two different methods were used: The PrestoBlue™ assay for metabolic activity and the CytoTox-ONE™ assay for membrane integrity of the cells. The PrestoBlue™ assay was performed with the L-929 cells based on ISO10993-5. In detail, cells (concentration as indicated above) were seeded in 100 µL per well in a 96-well plate without using the outer wells and treated in sextuplicates with polymers at different concentrations, ranging from 5 µg mL^−1^ to 200 µg mL^−1^ for 24 h. The medium was replaced by a 10 % (v/v) PrestoBlue™ solution in fresh culture medium, prepared according to the manufacturer’s instructions. Following an incubation at 37 °C for 45 min, the fluorescence was measured at *λ*_Ex_ = 570 / *λ*_Em_ = 610 nm. Non-treated control cells on the same plate were referred to as 100% viability. Values above 70 % were regarded as non-toxic. To assess the toxicity of polyplexes used for transfection, K-562 and HEK293T cells were seeded in 500 µL per well in a 24-well plate and treated with the polyplexes at N*/P 30 for 24 h prior to the PrestoBlue™ assay. The polyplexes were prepared as described above with isolated pKMyc pDNA and added to the cells diluting the polyplexes 1:10 in the cell culture medium. Data are expressed as mean ± SD of at least three independent determinations.

For determination of the release of lactate dehydrogenase (LDH) due to membrane disruption, the CytoTox-ONE™ assay (LDH-assay) was performed according to the manufacturer’s instructions following incubation of the cells with polyplexes as described above in 500 µL per well of a 24-well plate for 24 h. The supernatant was transferred to a new 96-well plate as a triplicate and allowed to cool down to room temperature for 30 min. Subsequently, the substrate mixture including assay buffer was added and incubated at room temperature for 10 min. The fluorescence intensity was measured at *λ*_Ex_ = 560 nm / *λ*_Em_ = 590 nm following the addition of the stop solution. For the positive control (100 % LDH release), cells were incubated with 0.2 % Triton X-100 for 30 min prior to analysis. Cells incubated with only pDNA were used as negative control (0 % LDH-release). The relative number of viable cells with intact membranes was calculated as follows (2):

2 $${\text{Viability}}/\% = 100 - \frac{{{{\text{F}}_{{\text{Sample}}}} - {{\text{F}}_0}}}{{{{\text{F}}_{{\text{Positive~control}}}} - {{\text{F}}_0}}}~ \cdot 100$$

Where F_Sample_, F_0_, and F_Positive control_ represent the fluorescence intensity of a given sample, medium without cells, and of the Triton X-100 treated cells, respectively.

### Transfection efficiency

Transfection studies were performed in HEK293T, K-562 and further leukemia cells. The cells were seeded in 500 µL per well in 24-well plates and treated with polyplexes at N*/P 30. The polyplexes were prepared as described above with isolated mEGFP-N1 pDNA and added to the cells diluting the polyplexes 1:10 in the cell culture medium for an incubation period of 24 h. For analysis via flow cytometry, HEK293T cells were harvested by transferring the supernatant to a 24-well plate, trypsinizing the cells and resuspending them in the respective supernatant again. Subsequently, fresh D10 was added, diluting the cell suspension 1:2. Half of the suspension was transferred to a 96-well plate for measurement, while the remaining cells were incubated for another 48 h. K-562 cells were harvested by resuspension and subsequent transfer of half of the cell suspension to a 96-well plate for measurement. For long-term transfection (72 h), the remaining cell suspension was split 1:2 by adding the same amount of fresh R10 and incubated for further 48 h.

For determination of transfection efficiency, cells were analyzed as described in the instrumentation section (Additional file 1). Viable cells showing EGFP signal higher than the mock control cells incubated with polyplexes of the respective polymer and pKMyc pDNA were gated as percentage of cells expressing EGFP and the relative mean fluorescence intensity (rMFI) of all viable cells was calculated in relation to the respective mock control. The experiments were performed at least three times and data are expressed as mean ± SD.

### Polyplex uptake

To study the uptake of polymers over time in HEK293T and K-562 cells, the cells were seeded in 500 µL per well in 24-well plates and treated with polyplexes at N*/P 30 for indicated time periods. The polyplexes were prepared as described above after labelling 1 µg pKMyc pDNA with 0.027 nmol YOYO-1 iodide. Subsequently, the polymer-pDNA-solutions were added to the cells, diluting the polyplexes 1:10 in cell culture medium. Following incubation, the HEK293T cells were harvested by trypsinization and resuspension in FC-buffer (Hanks’ Balanced Salt Solution, supplemented with 2 % FCS and 20 mM HEPES), while the K-562 cells were only resuspended. Trypan blue solution (0.4 %) was added to a final concentration of 0.04 % to quench fluorescence of polyplexes outside the cells. Cells were analyzed via flow cytometry or confocal laser scanning microscopy (CLSM) as described in the instrumentation section (Additional file 1). Viable cells showing YOYO-1 signal higher than the control cells, which were incubated with YOYO-1-pDNA only, were gated as percentage of cells that have taken up pDNA and the rMFI of all viable cells was calculated in relation to the control cells. The experiments were performed at least three times and data are expressed as mean ± SD.

### Calcein release assay

To determine the endosomal escape efficiency of the polymers, a calcein release assay was performed with HEK293T and K-562 cells. The cells were seeded in 500 µL per well in 24-well plates and treated with polyplexes at N*/P 30. Just before the addition of polyplexes, the non-cell-permeable dye calcein was added to the cells to give a final concentration of 25 µg mL^−1^. Following incubation for different time periods, the cells were washed via centrifugation at 250×*g* for 5 min. Prior to the washing step, the HEK293T cells were harvested by trypsinization and resuspension in FC-buffer, whereas the K-562 cells were only resuspended. Via flow cytometry, cells were analyzed as described in the instrumentation section (Additional file 1). Viable cells showing a calcein signal higher than the control cells incubated with calcein only were gated as percentage of cells that show strong calcein signal and the rMFI of all viable cells was calculated in relation to the control cells. The experiments were performed three times.

### Statistics

To determine the statistical significance, repeated measures analysis of variance (RM-ANOVA) was performed. If the RM-ANOVA revealed significant differences (*p* < 0.05), post-hoc analyses with a Bonferroni correction were applied. If not stated otherwise, statistically significant differences to the control were indicated with * for *p* < 0.05, ** for *p* < 0.01, and with *** for *p* < 0.001. All statistical analyses were performed with data of n ≥ 3 in Origin, Version 2018b (OriginLab Corporation, US). Further details can be found in the Additional file 1.

## Results and discussion

### Polymer synthesis and micelle formation

**Scheme 1:**
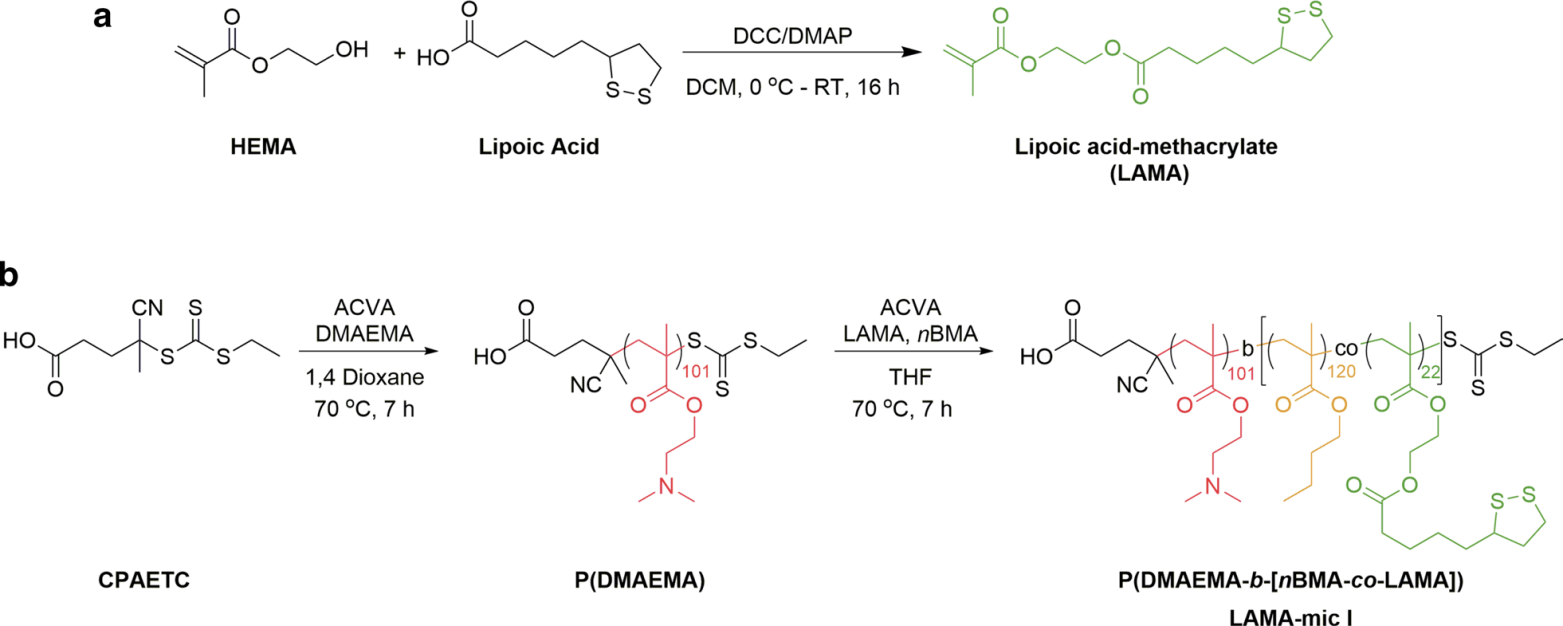
Synthetic routes. **a** Synthesis of the LAMA monomer via DCC/DMAP esterification coupling reaction. **b** A chain transfer agent, CPAETC, was used to synthesize a macro-CTA, PDMAEMA, via RAFT polymerization. The macro-CTA was then used to synthesize the block copolymer under same conditions

Lipoic acid has antioxidant potential and has been studied for treatment of different diseases. In addition, studies have shown that lipoic acid exhibits redox properties, thus, they can be used as a crosslinking agent when incorporated in micelles or nanoparticles [52]. Two approaches for incorporating functional groups into polymers include (i) coupling them to a monomer and (ii) post-modification of a polymer. In this study, we opted for the first method due to the advantages for quantification and analysis that are provided by this method. Lipoic acid-methacrylate (2-(methacryloyloxy)ethyl 5-(1,2-dithiolan-3-yl)pentanoate, LAMA), was synthesized by a N,N′-dicyclohexylcarbodiimide (DCC)/4-(dimethylamino)pyridine (DMAP) coupling reaction (Scheme 1a), which is a well-established method for esterification reactions [53].

The polymers were synthesized by RAFT polymerization with (4-cyano pentanoic acid)yl ethyl trithiocarbonate (CPAETC) as a chain transfer agent (CTA) and 4,4′-azobis(4-cyanovaleric acid) (ACVA) as the initiator. Firstly, a PDMAEMA homopolymer was synthesized to serve as a macro-CTA for block copolymer synthesis as well as a control polymer for biological studies (Scheme 1b). Previous research had demonstrated that homopolymers of DMAEMA as well as the acrylamide analogue dimethylamino ethyl acrylamide (DMAEAm) were less toxic than LPEI but exhibited decreasing viability of cells with increasing molar mass [50, 54]. For this reason, a target degree of polymerization (DP) of 100 was chosen for the cationic homopolymer and more importantly, to achieve desired spherical micelles of sizes less than 100 nm. The macro-CTA was subsequently chain-extended with LAMA and *n*BMA to form a block copolymer P(DMAEMA-*b*-[*n*BMA-*co*-LAMA]). *n*BMA was chosen because of its biological benefits, i.e. for the interaction with cell membranes [55]. While PDMAEMA was incorporated as the hydrophilic and pH-responsive segment, the LAMA monomer was statistically integrated into the hydrophobic block of *n*BMA. The overall composition (hydrophobic to hydrophilic) was optimized to facilitate the formation of spherical micelles. All polymerizations were carried out at 70 °C and stopped after 7 h to avoid high polydispersity due to dead chain formation. Monomer conversions were typically in the range of 65 to 70% for the homopolymers and 40 to 80 % of the subsequently extended block (determined via ^1^H NMR, Additional file 1: Table S3, Fig. S2). SEC analysis of the polymers showed narrow molar mass distributions (*Ð* = 1.17 for PDMAEMA and *Ð* = 1.19 for P(DMAEMA-*b*-[*n*BMA-*co*-LAMA]), Additional file 1: Fig. S1, S3).

**Fig. 1:**
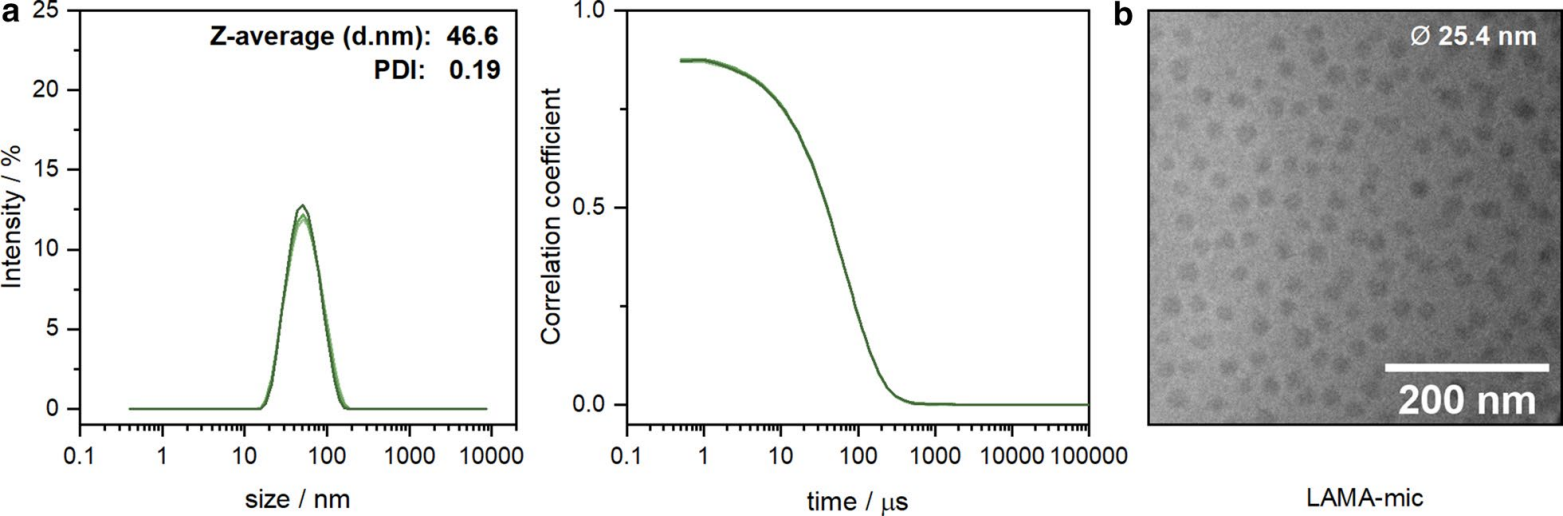
Morphology of assembled block copolymers. **a** DLS intensity and correlation plots showing unimodal distribution with an average size below 100 nm and small PDI. **b** Cryo-TEM image of the formulated micelles from solvent exchange method showing a homogenous spherical morphology

Micelles of the diblock copolymers P(DMAEMA_101_-*b*-[*n*BMA_120_-*co*-LAMA_22_]) (LAMA-mic), were formed by solvent-exchange followed by dialysis in water. DLS measurements were conducted to determine the hydrodynamic size of the formulated micelles (Fig. 1a, Additional file 1: Table S4). The results showed a monomodal distribution with an average hydrodynamic radius of 47 nm and a polydispersity index of 0.19. It is worth noting that the size below 100 nm is favorable for endocytic uptake [56]. Additionally, cryo-TEM measurements were conducted to investigate the morphology of the particles (Fig. 1b). The images showed micelles featuring a spherical morphology with rather homogeneous particle size distributions (Ø 25.4 ± 2.9 nm). The slightly smaller size measured by cryo-TEM compared to DLS is common due to the micellar shell and the hydrodynamic interactions measured by cryo-TEM showing little or no contrast.

### Polyplex formation and characterization

**Fig. 2:**
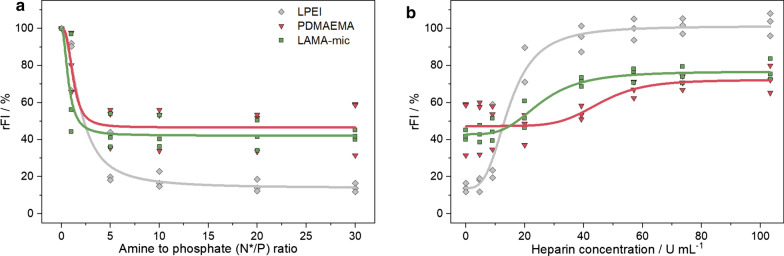
Polyplex formation and stability tests with pDNA. **a** EBA at different N*/P ratios in HBG buffer pH 7.4. **b** HRA of polyplexes at N*/P 30 in HBG buffer pH 7.4. **a + b** Dots represent individual replicates and lines represent the logistic fit to the respective data points (n = 3)

To investigate whether the targeted micelle, LAMA-mic, is able to bind genetic material such as pDNA, fluorescence-based assays as EBA and HRA were performed (Fig. 2). The EBA was conducted at different N*/P ratios (molar ratio of amines of the polymer to phosphates of the genetic material) with a constant amount of pDNA. A decrease in the fluorescence signal of the pDNA-EtBr solution upon addition of the polymers indicated a successful formation of polyplexes due to the displacement of the EtBr from the pDNA. To investigate the stability of the polyplex, the polyanionic polysaccharide heparin was added to polyplexes of N*/P 30. Due to its negative charge, heparin can bind to the cationic moiety, replace the pDNA and EtBr can thus intercalate again into the pDNA, resulting in an increasing fluorescence intensity (FI). All tested polymers were shown to decrease the relative FI (rFI) in the EBA but to a different extent. The binding efficiency of the LAMA-mic (42 ± 3 % rFI) was comparable to that of the PDMAEMA homopolymer (50 ± 16 % rFI) demonstrating that the architecture has no influence here. However, the EtBr displacement with the LAMA-mic was not as strong as with LPEI (14 ± 2 % rFI). A gel retardation assay (GRA) indicated a complete retention of the pDNA in polyplexes at N*/P ≥ 10 for all polymers in contrast to the migration of pure pDNA (Additional file 1: Fig. S6). Since the increase in EtBr fluorescence intensity is based on an intercalation between the hydrophobic base pairs of the double helix, [57, 58] the EtBr displacement in case of the LAMA-mic and PDMAEMA may be distorted by the presence of more hydrophobic monomers (for logP values refer to Additional file 1: Table S6) and uncharged cationic moieties at pH 7.4, respectively.

Regarding polyplex stability, the influence of the micellar composition is more pronounced. While LPEI required only 18 U mL^− 1^ heparin to release 70 % of the pDNA, the LAMA-mic required double and PDMAEMA nearly four times the amount of heparin, indicating an increased stability of the latter against anionic antagonists, which was also shown for other micellar systems [55]. The PDMAEMA-based systems reached plateaus around 70 % rFI. This could be caused by hydrophobic interactions with the pDNA, e.g. by the hydrophobic monomers or uncharged DMAEMA moieties at pH 7.4, which are less influenced by an anionic reagent like heparin. Overall, the LAMA-mic exhibits strong pDNA binding properties required for efficient gene carriers.

### Cytotoxicity

**Fig. 3:**
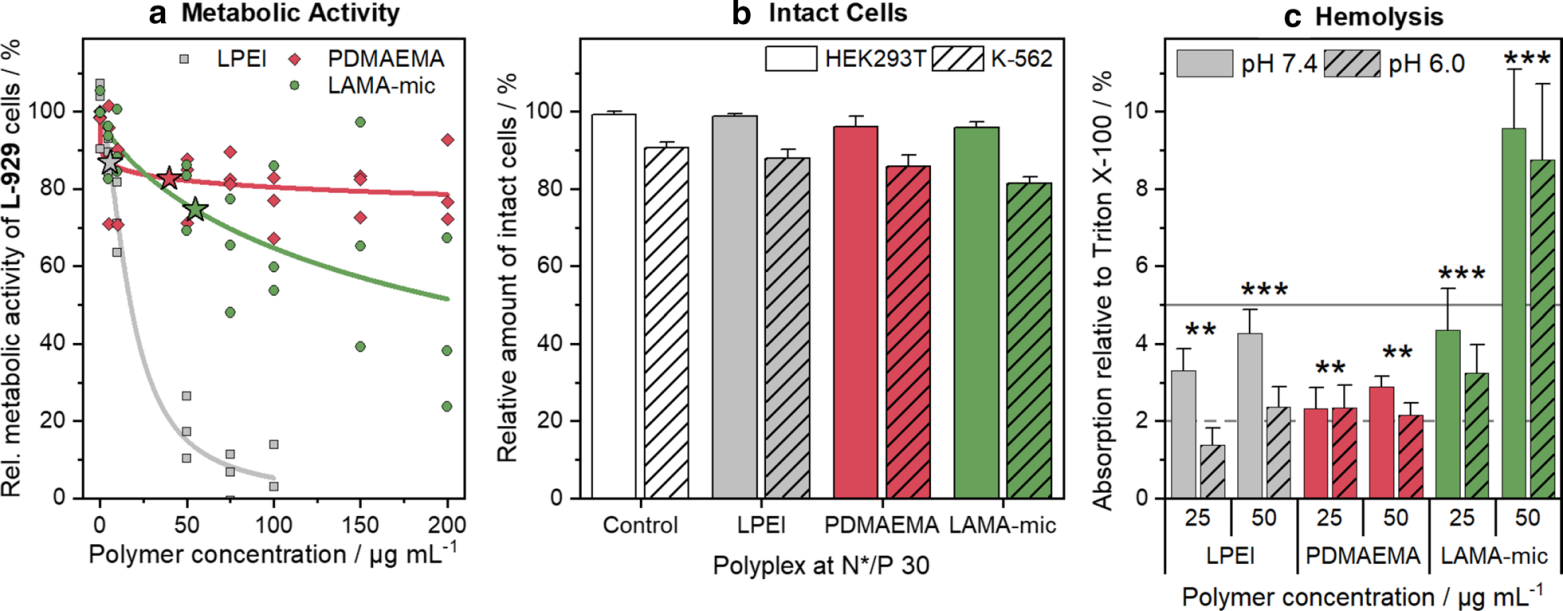
Toxicity of LAMA-mic in different cell lines. **a** PrestoBlue™ assay in L-929 cells following incubation with respective polymers at indicated concentrations for 24 h. Dots represent values of single repetitions and lines represent logistic fit functions (n = 3). Stars indicate polymer concentrations used for N*/P 30 in transfection assays. **b** LDH-assay in HEK293T and K-562 cells following incubation with polyplexes of respective polymers and pDNA at N*/P 30 for 24 h. Values were calculated relative to the positive control Triton X-100 (100 % LDH release ≙ 0 % viability) and represent mean ± SD (n = 3). **c** Hemolysis as the amount of released hemoglobin calculated relative to 1 % Triton X-100 as positive control (100 % hemolysis). Human erythrocytes were washed and incubated with polymers at indicated concentrations in PBS of different pH values without serum. Values represent mean ± SD (n = 3) and are classified as slightly hemolytic between 2% and 5%, and as non- or hemolytic if lower or higher than 2% or 5%, respectively. Regarding significant differences, the main effects of the treatment were determined since there was no significant interaction of pH value and treatment

Since the presence of cationic charges and hydrophobic side chains in polymers might be problematic for cells, [36, 59] the LAMA-mic was investigated regarding its cytotoxic effects in different cell lines. To assess the metabolic activity of the cells, the PrestoBlue™ assay was performed based on ISO10993-5 with L-929 cells (Fig. 3a). The cells incubated with LAMA-mic and the PDMAEMA homopolymer showed less cytotoxicity at higher concentrations than the commercial LPEI which served as control. At concentrations equal to N*/P 30, which was later used to investigate the transfection efficiency, incubation with polymers resulted in cell viability above 70 % in all cases, indicating good viabilities. The metabolic activity was further investigated in HEK293T and K-562 cells applying polyplexes at N*/P 30 (Additional file 1: Fig. S7A). Especially in HEK293T cells, the LAMA-mic exhibited lower metabolic activity (57 ± 8 % viable cells), although the cells did not appear to be dead when observed by light microscopy (Additional file 1: Fig. S8). Upon treatment with the LAMA-mic, HEK293T cells exhibited a rounded shape, formed spheroids, and started to detach. This phenomenon was observed for other polymers as well, [60] but more detailed investigations are required to understand the principle mechanism for this material, which was not the focus of this study.

To ensure that the decreased metabolic activity was not caused by a removal of viable yet detached cells during the washing steps or by the assay reagent not reaching the center of the HEK293T spheroids, other assays employing different cytotoxicity mechanisms were performed. The influence of the polyplexes on the membrane integrity was investigated using the LDH-assay and propidium iodide staining in combination with flow cytometry (Fig. 3b, Additional file 1: Fig. S7B, C). In both assays, an entering of the reagent into the cells is not necessary and the supernatant is measured either pure (LDH-assay) or together with the single cell suspension by re-addition after trypsinization (flow cytometry). The LDH-assay is based on resazurin, measuring the activity of the enzyme LDH after its release into the medium if the membrane integrity of the cells was destroyed. In flow cytometry, on the other hand, dead cells can be identified due to their changes in shape and granularity in the FSC/SSC plot. Propidium iodide staining was used as a further identification of dead cells to adjust the analysis parameters. HEK293T cells treated with the LAMA-mic exhibited viabilities above 90 % with both methods, LDH-release and FSC/SSC, whereas K-562 cells showed nearly the same viability as observed in the PrestoBlue™ assay (70 to 80% viable cells). These results indicate that the LAMA-mic is not or only slightly influencing the membrane integrity of HEK293T and K-562 cells, respectively, and that the spheroid formation of the HEK293T cells could be a result of the LAMA-mic’s influence on the interaction between HEK293T cells and the surface of the cultivation vessel rather than of toxic effects.

The impact of the polymers on membranes was investigated using human erythrocytes in hemolysis (Fig. 3c) and aggregation assays (Additional file 1: Fig. S9). Following serum removal, the cells were resuspended in PBS of pH values present in blood/cytoplasm (pH 7.4) or endosomal compartments (pH 6) and incubated with the polymers for 1 h, followed by centrifugation and absorption measurement of the released hemoglobin in the supernatant. Whereas LPEI and PDMAEMA were only slightly hemolytic (≤ 4.4 % hemoglobin release), the LAMA-mic showed doubled hemolytic activity at 50 µg mL^− 1^, indicating a contribution of the micellar hydrophobic core or the locally increased amount of cationic charges due to micellar architecture to membrane interaction. With the exception of LPEI, there was no significant influence of pH value on hemolysis (*p* = 0.06).

The observed low toxicity for PDMAEMA is consistent with results of previous studies [54, 61]. However, the increased membrane interactions of the LAMA-mic are not too surprising as the integration of hydrophobic moieties (*e.g. n*BMA) is known for this effect [62, 63].

### Transfection efficiency

**Fig. 4:**
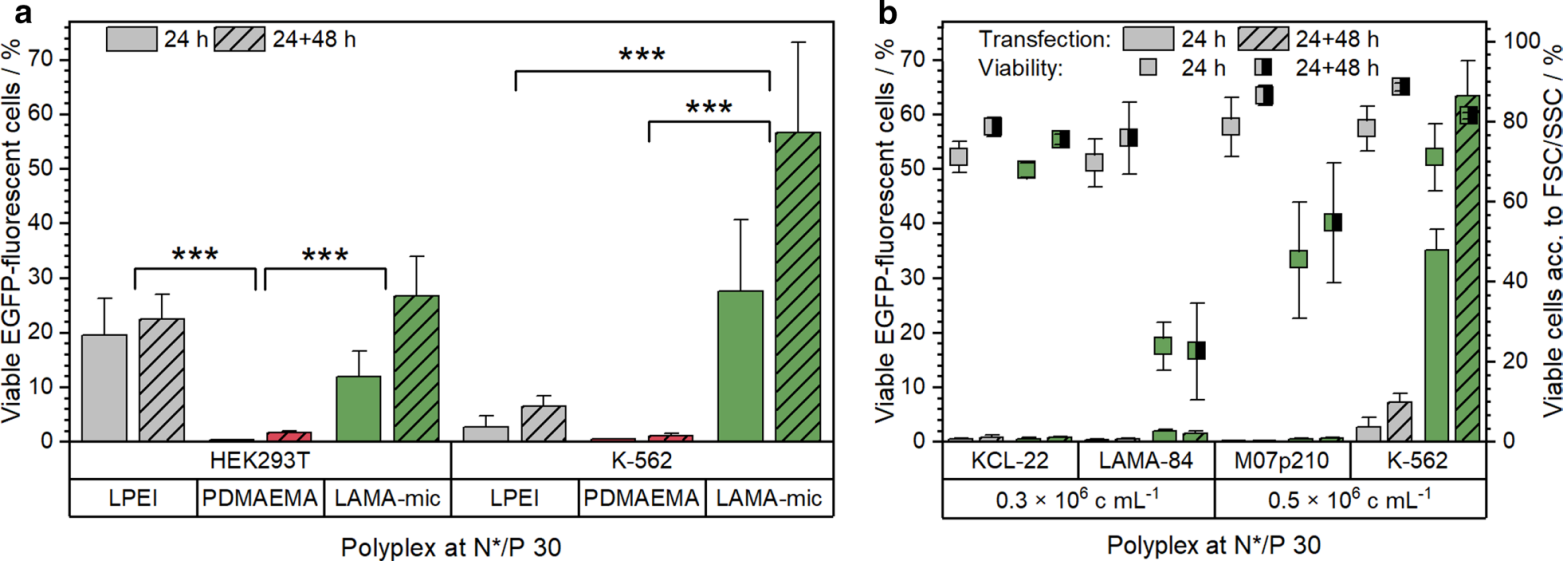
Transfection efficiency of polymers in different cell lines. **a** EGFP expression of viable cells was analyzed via flow cytometry following incubation of cells with polyplexes of mEGFP-N1 pDNA and polymers at N*/P 30 in respective growth medium (D10 or R10 with 10 mM HEPES) for 24 h or for 24 h followed by splitting of cells and medium and further incubation for 48 h. Values represent mean ± SD (n ≥ 3). Regarding significant differences, only the main effects of the treatment were determined since there was no significant interaction of incubation time and treatment. **b** EGFP expression of viable cells in AML or CML cell lines treated as described above and seeded as indicated below the graph. Viability was determined according to the FSC/SSC scatter plot of flow cytometry. Values represent mean ± SD (n ≥ 3)

The gene delivery to suspension cells is for unresolved reasons less efficient in comparison to adherent cells [8]. Therefore, the transfection efficiency of the polymers was investigated by treating HEK293T and K-562 cells with polyplexes of polymers and pDNA encoding for EGFP at the optimal N*/P ratio 30. Following incubation (24 h or 24 h with additional 48 h in growth media), cells were analyzed via flow cytometry regarding the amount of viable and EGFP-positive cells (Fig. 4a) and the relative mean fluorescence intensity (rMFI) of all viable single cells (Additional file 1: Fig. S10).

In both cell lines, the LAMA-mic showed an increased number of EGFP-positive cells but with peculiar differences. Whereas in HEK293T cells the transfection efficiency of LAMA-mic and LPEI were comparable after 24 h, the LAMA-mic outperformed LPEI by more than seven times in K-562 cells (29 ± 12 % vs. 2 ± 2 % viable EGFP-positive cells, *p* < 0.001). Interestingly, the transfection efficiency of the LAMA-mic increased almost twofold in both cell lines following incubation in growth media for further 48 h, although being only significant in K-562 cells (*p*(HEK293T) = 0.15, *p*(K-562) = 0.03). This could indicate a slower, continuous transfection mechanism (i.e. polyplex uptake and endosomal escape) for the LAMA-mic, whereas LPEI could reach its maximum already after 24 h. In contrast, the homopolymer PDMAEMA showed nearly no transfected cells in both cell lines (< 2 % EGFP-positive cells). This is in accordance to previous studies of PDMAEMA [54, 61, 64]. Successful transfections with PDMAEMA could only be observed at higher molecular weight and/or higher pDNA concentration [65, 66].

Since the LAMA-mic results in a remarkably efficient gene expression in K-562 cells, even without the addition of transferrin and although these cells are considered to be difficult to transfect, the following sections will discuss, which parameter can be responsible for this. One reason could be the altered metabolism of these cells due to the generation of the *BCR-ABL* oncogene encoding for the BCR-ABL fusion protein [67, 68]. Hence, the transfection efficiency of the LAMA-mic was investigated in the additional BCR-ABL positive CML cell lines LAMA-84 and KCL-22, and in the AML cell line M07p210 expressing the fusion protein as well. As shown in Fig. 4b, none of the cell lines could be transfected with the same conditions which were successful in K-562 cells. On the contrary, the LAMA-84 and M07p210 cells showed severe toxicity following incubation with the LAMA-mic implying that BCR-ABL is not the crucial point for the transfection efficiency of the LAMA-mic, and that there must be other/additional parameters whose determination will need more profound investigations, e.g. regarding the influence of differences in signaling and metabolism of the cell lines [69] on the transfection efficiency.

### Transfection mechanism of LAMA-mic

**Fig. 5:**
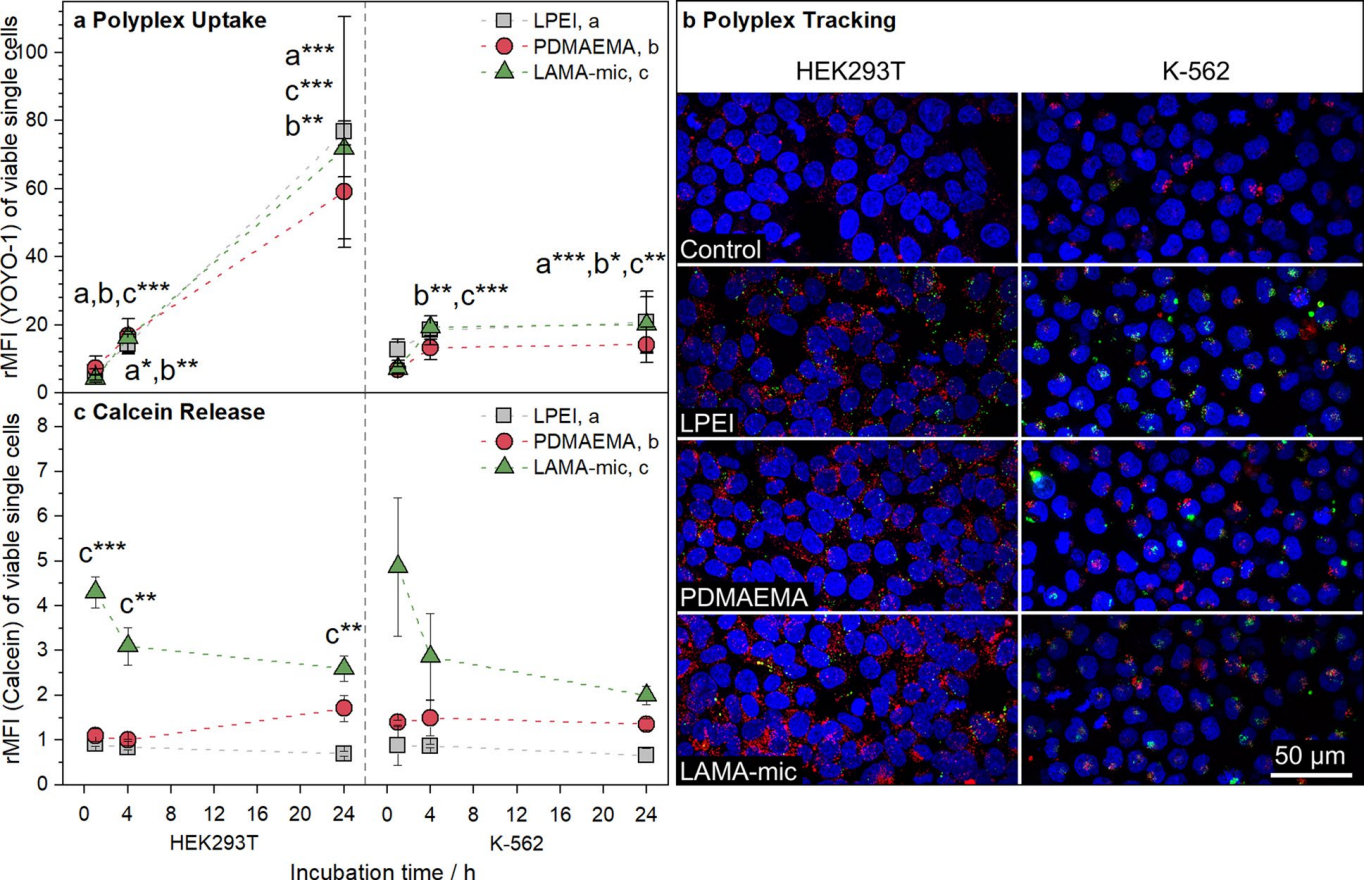
Investigation of the gene delivery process. **a** Cellular internalization of LAMA-mic polyplexes in different cell lines. Cells were incubated with polyplexes of polymers and YOYO-1-labeled pDNA at N*/P 30 and analyzed via flow cytometry. Cells incubated with labeled pDNA served as control (rMFI = 1). Values represent mean ± SD (n = 3). Asterisks indicate significant differences of LPEI (a), PDMAEMA (b), LAMA-mic (c) to the control of the respective time point. **b** Cellular internalization of polyplexes investigated via CLSM following incubation of cells with polyplexes of polymers and YOYO-1-labeled pDNA at N*/P 30 for 1 h (n ≥ 2). Endolysosomes were stained with LysoTracker^TM^ Red (red), nuclei were stained with Hoechst33342 (blue) and YOYO-1 fluorescence (green) was quenched with trypan blue. Maximum intensity projection was used to display polyplexes in every plane of the cell. **c** Endosomal escape of LAMA-mic polyplexes was detected by the non-permeable dye calcein and analyzed via flow cytometry relative to the calcein control (rMFI = 1). Values represent mean ± SD (n = 3). If not stated otherwise, asterisks indicate significant differences to the control of the respective time point. Since there was no significant interaction of incubation time and treatment for K-562 cells, the main effects of the treatment were determined with only LAMA-mic showing a significant difference to the control (*p* < 0.001)

For elucidation of the LAMA-mic’s high transfection efficiency in the suspension cell line K-562, two crucial cellular issues of gene delivery, namely, the cellular uptake and the endosomal release, were studied in more detail. To assess the polyplex uptake, K-562 and HEK293T cells were incubated with YOYO-1 labelled polyplexes at N*/P 30 for different time periods and analyzed via flow cytometry regarding the amount of YOYO-1 positive cells (Additional file 1: Fig. S12A) and rMFI of all viable single cells or via CLSM regarding the intracellular distribution of the polyplexes (Fig. 5a, b and S15-16). For CLSM studies, the cells were additionally labeled with LysoTracker^TM^ Red for endolysosomes and Hoechst 33342 for nuclei. Both cell lines show YOYO-1 positive cells after 1 h already with some of the polyplexes located in acidic compartments. It is remarkable that no significant differences between the polymers were observed regarding the rMFI in flow cytometry. To the contrary, the uptake patterns of the cell lines were found to be different. Only PDMAEMA showed slightly lower rMFI values in both cell lines. In HEK293T cells, a time-dependent uptake was found for all polyplexes, whereas in K-562 cells the uptake reached a constant level after 4 h. After 1 h and consistent with the CLSM images, the uptake in K-562 cells was slightly higher than in HEK293T cells, whereas after 24 h, the uptake in HEK293T was almost four times as high as in K-562 cells. A low endocytosis rate by suspension cells was also observed in other studies [20, 70]. This may also be associated with increased exocytosis, where the uptaken polyplexes are removed at a rate comparable to the rate they are taken up. As these results do not correlate to the observed transfection efficiencies, the observed differences of cellular uptake are not a critical aspect for the LAMA-mic in K562 cells.

Moreover, the endosomal escape was investigated using the calcein release assay. Calcein is a non-membrane-permeable dye taken up by endosomes. If a polymer is able to escape the endosome by attacking the endosomal membrane, calcein can be released into the cytoplasm, causing a diffuse fluorescence pattern, which can be detected via flow cytometry, e.g. in an alteration in the pattern of the FITC channel (Additional file 1: Fig. S14). Therefore, the cells were incubated with calcein and polyplexes at N*/P 30 for different periods of time and were analyzed via flow cytometry (Additional file 1: Fig. S12B and Fig. 5b). In contrast to the pDNA uptake, the endosomal release showed almost no dependence on the cell line but on the polymer used. In both cell lines the LAMA-mic exhibited a significantly better release of calcein (*p* < 0.01), in particular during the first hours (1–4 h), indicating the potential of block copolymers for endosomal release also in difficult to transfect cell lines.

### Influence of lipoic acid on stability and transfection efficiency

To investigate the role of lipoic acid for the improved transfection efficiency of the LAMA-mic in detail, an additional set of polymers was prepared comprising two polymers without lipoic acid, P(DMAEMA_89_-*b*-*n*BMA_68_) and P(DMAEMA_89_-*b*-[*n*BMA_92_-*co*-HEMA_17_]) (see Additional file 1: Table S3). The first resembled an amphiphilic block copolymer, which lacks the LAMA moiety, while the second was synthesized to mimic a hydrolyzed and cleaved lipoic acid from the original LAMA copolymer. Another version of the LAMA-mic (P(DMAEMA_89_-*b*-[*n*BMA_101_-*co*-LAMA_19_]) copolymer, LAMA-mic II) was synthesized for the comparison of all polymers with the same macro-CTA. The polymeric micelles (BMA-mic, HEMA-mic and LAMA-mic II) were spherical, unimodal and similar in size (Additional file 1: Table S4, Fig. S4, S5).

To investigate the influence of lipoic acid on the micelle stability, the critical micelle concentration (CMC) was determined using Nile Red encapsulation as a fluorescence probe (Additional file 1: Fig. S18) [71]. The results showed comparable CMC values for all micelles below the concentrations used for cell experiments (LAMA-mic II: 29, HEMA-mic: 30, BMA-mic: 26 µg mL^− 1^) indicating the presence of mainly micelles with comparable physicochemical properties during transfection (Additional file 1: Table S5). Additionally, the polyplex stability was investigated by DLS following dilution of the respective polyplexes to concentrations used for transfection (Additional file 1: Fig. S17, Table S4). Although the sizes of the micelles hardly changed, the correlation coefficient of lipoic acid free HEMA- and BMA-mic decreased, whereas it remained constant in the case of LAMA-mic II polyplexes, indicating a synergistic stabilizing effect of lipoic acid and the hydrophobic monomers.

**Fig. 6:**
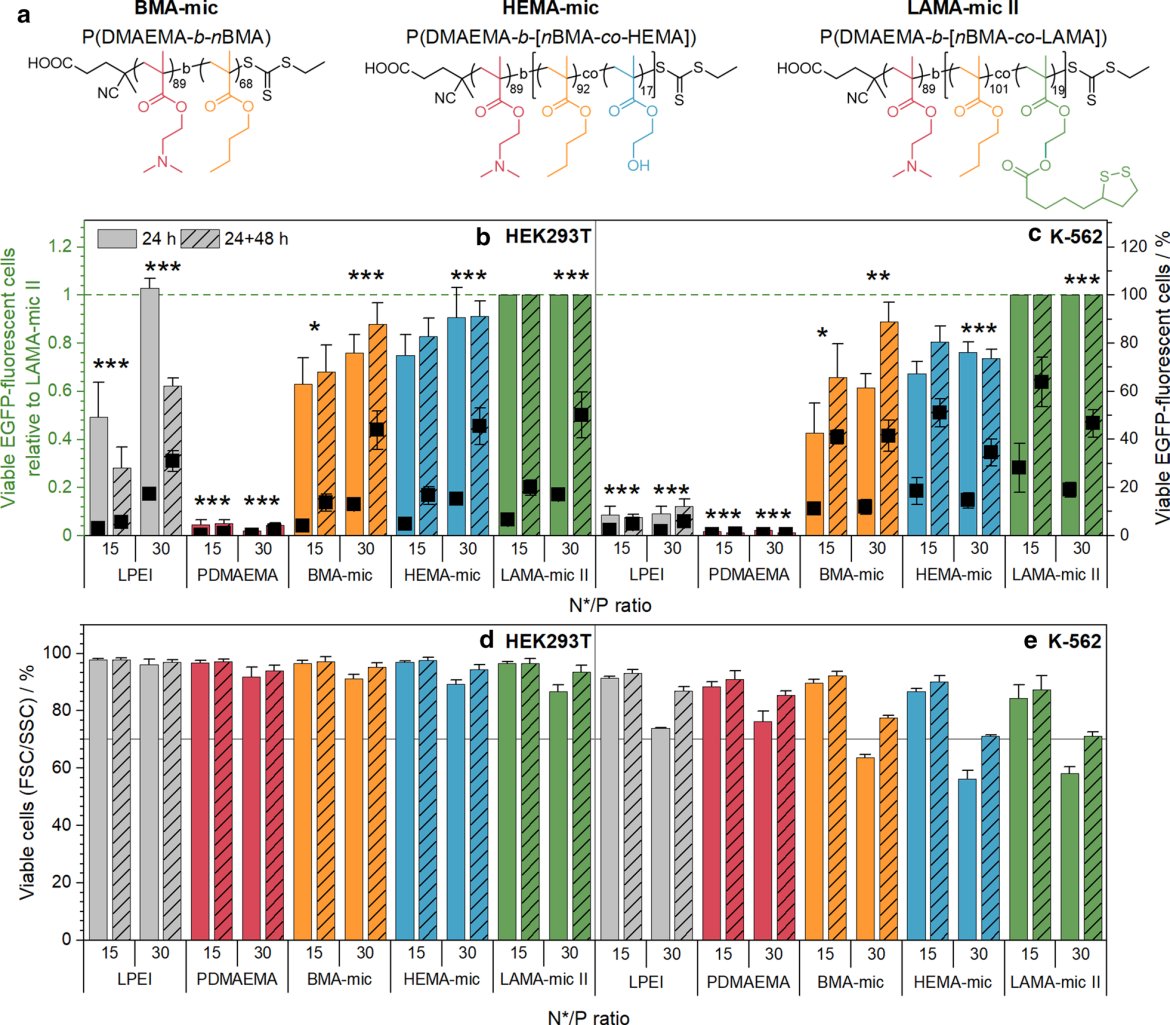
Transfection efficiency and toxicity of LAMA-mic compared to control micelles. **a** Overview of the structures of the additional polymer set. **b** + **c** EGFP expression of viable cells was analyzed via flow cytometry following incubation of cells with polyplexes of mEGFP-N1 pDNA and polymers at N*/P 30, in respective growth medium (D10 or R10 with 10 mM HEPES) either for 24 h or for 24 h followed by splitting of cells and medium and further incubation for 48 h. Values represent mean ± SD (n = 3) of EGFP-positive cells relative (columns) and not relative (squares) to the respective LAMA-mic treatment. **d** + **e** Viability was determined according to the FSC/SSC scatter plot of flow cytometry. Values represent mean ± SD (n = 3). **b** + **d** HEK293T cells. **c** + **e** K-562 cells. Regarding significant differences, the main effects of the treatment were determined since there was no significant interaction of incubation time and treatment. *: significant difference to LAMA-mic II, N*/P 15 (*p* < 0.05). **: significant difference to LAMA-mic II, N*/P 15 (*p* < 0.01). ***: significant difference to LAMA-mic II, N*/P 15 (*p* < 0.001)

With suitable polymers available, HEK293T and K-562 cells were incubated and analyzed as described before with the corresponding polyplexes at N*/P 15 and 30 (Fig. 6a, b). When comparing the different micelles, the micelles without lipoic acid were also able to transfect HEK293T cells, although to a slightly lesser extent. In K-562 cells, the transfection efficiency of the LAMA-mic II polyplexes at N*/P 15 was significantly higher than the polyplexes of the other micelles at N*/P 30 (HEMA-mic: 22 ± 11 % points, ppt; *p* < 0.001; BMA-mic: 19 ± 4 ppt, *p* = 0.002) indicating an increase in efficiency by the incorporation of lipoic acid into micelles. The FSC/SSC plot of the flow cytometer was used to investigate the cytotoxicity of the polyplexes at the applied concentrations (Fig. 6c, d). Whereas the HEK293T cells showed no viabilities less than 89 % in all cases, the K-562 cells exhibited decreased viability, in particular at N*/P 30 after 24 h (BMA-mic: 64 ± 1%, HEMA-mic: 56 ± 3 %, LAMA-mic II: 58 ± 2 % viable cells). Nevertheless, the K-562 cells could recover as indicated by an increase in viability to more than 70 % viable cells following splitting and incubation for further 48 h, which is linked to higher transfection efficiency at N*/P 15, especially for LAMA-mic II (black squares in Fig. 6a, b). For HEK293T cells, however, the increase in efficiency was less pronounced compared to the K-562 cells.

In summary, all three micelle systems represent promising candidates for efficient gene delivery to K-562 cells with the higher transfection efficiency of the LAMA-mic II indicating a synergistic effect for the lipoic acid and the architecture.

## Conclusions

Since the cells of our immune system and blood are known to be hard to transfect or modulate, diseases like CML still suffer problems like drug resistance or poor treatment response. Polymeric nanocarriers could be a solution to enhance interaction with blood cells and to increase the intracellular concentration of active agents. Therefore, a well-defined lipoic acid containing diblock copolymer, P(DMAEMA_101_-*b*-[*n*BMA_124_-*co*-LAMA_22_]), was synthesized *via* RAFT polymerization and formulated into defined spherical micelles (LAMA-mic) to investigate its transfection efficiency of pDNA in different cell lines, including the CML cell line K-562. The LAMA-mic is able to bind and release the genetic cargo, caused low toxicity at used concentrations and exhibited transfection efficiencies comparable to that of the commercial control LPEI in HEK293T cells. Remarkably, whereas LPEI showed almost no transfection in K-562 suspension cells, the LAMA-mic exhibited an increase in EGFP-positive cells by more than sevenfold. Compared to the homopolymer PDMAEMA, the 54-fold increase in transfection efficiency was even more impressive and demonstrated the impact of the polymer design and/or architecture. This transfection efficiency does not seem to depend on the BCR-ABL fusion protein as a crucial point, since no other tested CML or AML cell line with this fusion protein could be transfected. Further mechanistic studies were performed regarding endosomal escape and polyplex uptake kinetics, which however could not be correlated to the performance of the polymers. In contrast to LPEI, the LAMA-mic showed good endosomal escape in both cell lines, pointing to potentially different escape mechanisms. The polymers did not show any effect on cellular uptake, although the cell line itself made a difference.

To investigate the effect of the lipoic acid functionality on transfection efficiency, two precursor polymers without the LAMA monomer, BMA-mic and HEMA-mic, were synthesized and formulated into micelles of comparable sizes and CMCs. It was found that the incorporation of lipoic acid into the core of a hydrophobic-cationic micelle enhances its gene delivery efficacy, especially in the difficult-to-transfect K-562 suspension cells. However, the influence of the architecture in general was more pronounced, as all micelles showed good efficiencies. Therefore, the LAMA-mic represents a promising nanocarrier system for gene delivery in hard-to-transfect blood cells.

## Supplementary information

**Additional file 1.** Description of further methods and results.

## Data Availability

The datasets used and/or analyzed during the current study are available from the corresponding author on reasonable request. Supporting information available: Material, additional methods and results, Additional file 1: Fig. S1–S18, Tables S1–S6.
