# Supplementary material for: Improved gene delivery to K-562 leukemia cells by lipoic acid modified block copolymer micelles
Source: J Nanobiotechnology. 2021 Mar 6;19:70. doi: 10.1186/s12951-021-00801-y (PMC7936509; doi:10.1186/s12951-021-00801-y)
Supplement: Supplementary file 1 — Additional file 1. Description of further methods and results. [file 12951_2021_801_MOESM1_ESM.pdf]

Supporting Information:

Improved gene delivery to K-562 leukemia cells by lipoic acid modified block copolymer micelles

Friederike Richter,[a,†] Prosper Mapfumo,[a,†] Liam Martin,[a] Jana I. Solomun,[a] Franziska Hausig,[a] Jochen J. Frietsch,[b] Thomas Ernst,[b] Stephanie Hoeppener,[a,c] Johannes C. Brendel,[a,c] Anja Traeger[a,c]*

[a]Laboratory of Organic and Macromolecular Chemistry (IOMC), Friedrich Schiller University Jena, Humboldtstrasse 10, 07743 Jena, Germany.

[b]Klinik für Innere Medizin II, Abteilung Hämatologie und Internistische Onkologie, Universitätsklinikum Jena, Am Klinikum 1, 07747 Jena, Germany.

[c]Jena Center for Soft Matter (JCSM), Friedrich Schiller University Jena, Philosophenweg 7, 07743 Jena, Germany.

[†]Authors contributed equally

*Correspondence to A. Traeger (anja.traeger@uni-jena.de)

List of Tables.

Table S1. Amount of different substances used for polymerization of polymers. 8
Table S2. Kinetic cycle protocol for automated heparin addition by the microplate reader........ 10
Table S3. Summary of polymer characterization. 17
Table S4. Summary of micelle characterization. 17
Table S5. Polymer concentrations in different assays. 20
Table S6. Computational analysis of monomer hydrophobicity by Molinspiration. 21

List of Figures.

Figure S1. Characterization of $PDMAEMA_{101}$ and $PDMAEMA_{89}$. 14
Figure S2. NMR results. 15
Figure S3. Kinetic results. 16
Figure S4. DLS measurements of the micelles. 18
Figure S5. Original cryo-TEM images of micelles. 19
Figure S6. Gel retardation assay. 20
Figure S7. Cytotoxicity of polymers in HEK293T and K-562 cells. 21
Figure S8. Spheroid formation in different cell lines. 22
Figure S9. Interaction of polymers with erythrocyte membranes. 22
Figure S10. Transfection efficiency of LAMA-mic in different cell lines. 23
Figure S11. Gating strategy for pDNA transfection using the example of 24 h incubation. 24
Figure S12. Investigation of the gene delivery process. 25
Figure S13. Gating strategy for polyplex uptake using the example of 4 h incubation. 26
Figure S14. Gating strategy for calcein release using the example of the 4 h incubation. 27
Figure S15. CLSM study of polyplex uptake in K-562 cells. 27
Figure S16. CLSM study of polyplex uptake in HEKT293T cells. 28
Figure S17. DLS measurement of the micelles at different concentrations. 29
Figure S18. CMC determination. 30

ADDITIONAL METHODS

Materials.

2-(Dimethylamino)ethyl methacrylate (DMAEMA) (98%), 2-Hydroxyethyl methacrylate (HEMA) (99%), *n*-Butyl methacrylate (*n*BMA) (99%), N,N′-Dicyclohexylcarbodiimide (DCC) (99%), 4-(Dimethylamino)pyridine (DMAP) (99%) and Nile Red (98%) were obtained from Sigma-Aldrich (Germany) and used as received. α,α′-Azoisobutyronitrile (AIBN) was obtained from Sigma-Aldrich and recrystallized from methanol. (4-cyano pentanoic acid)yl ethyl trithiocarbonate (CPAETC) and 2-(methacryloyloxy)ethyl 5-(1,2-dithiolan-3-yl)pentanoate (LAMA) were synthesized according to previous literature.[1, 2]

For biological studies, following substances were ordered from suppliers in brackets: cell culture media and supplements (Biowest, France), fetal calf serum (FCS, Capricorn Scientific, Germany), PrestoBlue^{TM} solution, YOYO^{TM}-1 iodide (Life Technologies, Thermo Fisher, Germany), trypsin-EDTA-solution, Triton X-100, 0.4% trypan blue solution and Hanks' balanced salt solution, calcein (Sigma-Aldrich), 1% ethidium bromide solution (EtBr, Carl Roth, Germany), heparin sodium salt from porcine intestinal mucosa (Alfa Aesar), linear poly(ethyleneimine) (LPEI, M_w = 25 kg mol^{-1}) and branched PEI (BPEI, M_w = 10 kg mol^{-1}, Polysciences, Germany), Green Gel Loading Buffer and High Range DNA Ladder (Jena Bioscience, Germany). mEGFP-N1 was a gift from Michael Davidson (Addgene plasmid #54767; http://n2t.net/addgene:54767; RRID: Addgene_54767). pKMyc was a gift from Ian Macara (Addgene plasmid #19400; http://n2t.net/addgene:19400; RRID: Addgene_19400).

Instruments.

Nuclear magnetic resonance (NMR) spectroscopy. ^{1}H NMR (300 MHz) and DEPT ^{13}C (75 MHz) spectra were recorded on a Bruker AC 300 MHz spectrometer at 300 K. The delay time (d1) was set at 1 s for ^{1}H NMR and 2 s for DEPT ^{13}C. Chemical shifts (δ) are reported in ppm.

Size exclusion chromatography (SEC). SEC was conducted on one of two instruments. Dimethylacetamide (DMAc)-SEC was conducted using an Agilent 1200 series instrument equipped with differential refractive index (DRI) and UV/VIS (DAD) detector. The liquid chromatography system used 1 × PSS GRAM 30 Å column (300 × 0.8 mm, 10 µm particle size) and 1 × PSS GRAM 1000 Å column (300 × 0.8 mm, 10 µm particle size). The DMAc eluent contained 0.21 wt.% LiCl as additive. Samples were run at 1 mL min^{-1} at 40 °C. Analyte samples were filtered through a polytetrafluoroethylene (PTFE) membrane with 0.45 µm pore size prior to injection. Poly(methyl methacrylate) (PMMA) narrow standards (PSS) were used to calibrate the SEC system. The measurements in chloroform were carried out on a Shimadzu system (Shimadzu Corp., Kyoto, Japan) equipped with a SCL-10A VP system controller, a SIL-10AD VP auto sampler, a LC-10AD VP pump, a RID-10A RI detector, a CTO-10A VP oven and a PSS SDVguard/lin S column (5 mm particle size). A mixture of chloroform/iso-propanol/triethylamine (94/2/4 vol%) was used as an eluent at a flow rate of 1 mL min^{-1} and an oven temperature of 40 °C. PMMA standards (400-100,000 gmol^{-1}) were used to calibrate the system. Experimental $M_{n,SEC}$ and $Đ$ (M_w/M_n) values of synthesized polymers were determined using PSS WinGPC UniChrom GPC software.

Flow cytometry. Flow cytometry was conducted on the CytoFlex S by Beckman Coulter GmbH, Germany. For each experiment, 10^4 cells per sample were analyzed regarding their viability in the

forward and sideward scatter (FSC/SSC) plot and their fluorescence at $\lambda_{Ex} = 488$ with a 525 nm bandpass filter, since all employed stains (YOYO-1, EGFP, calcein) were green fluorescent.

Microplate reader. Fluorescence intensity measurements for EBA, HRA, PrestoBlue™ and LDH assays as well as absorption measurements for hemolysis and aggregation assays were performed on the Infinite M200 PRO microplate reader (Tecan, Germany) with λ_{Ex} / λ_{Em} used as indicated in the respective method sections and gain set to optimal.

Detailed Monomer/Polymer Synthesis and Characterization.

Synthesis of lipoic acid methacrylate (2-(Methacryloyloxy)ethyl 5-(1,2-dithiolan-3-yl)pentanoate, LAMA). Lipoic acid (2.77 g, 1.34×10^{-2} moles), 2-Hydroxyethyl methacrylate (HEMA) (1.66 g, 1.27×10^{-2} moles) and 4-(Dimethylamino)pyridine (DMAP) (1.02 g, 8.37×10^{-3} moles) were dissolved in Dichloromethane (22 mL) in a round bottom flask and cooled under stirring over ice for approx. 10 min. N,N′-Dicyclohexylcarbodiimide (DCC) (3.36 g, 1.62×10^{-2} moles) dissolved in Dichloromethane (22 mL) was added dropwise to the mixture. The reaction mixture was cooled with ice for 30 min and then left at room temperature overnight. The crude mixture was dried under reduced pressure and then a flash column chromatography was performed to purify the product (hexane 60: ethyl acetate 40). ^{1}H NMR (300 MHz, Chloroform-*d*) δ 6.14 (s, 1H), 5.61 (s, 1H), 4.35 (s, 4H), 3.64 – 3.47 (m, 1H), 3.26 – 3.05 (m, 2H), 2.55 – 2.41 (m, 1H), 2.36 (t, J = 7.4 Hz, 2H), 1.98 – 1.83 (m, 4H), 1.78 – 1.58 (m, 4H), 1.56 – 1.41 (m, 2H). ^{13}C NMR (75 MHz, Chloroform-*d*) δ 173.16, 167.06, 135.93, 126.00, 62.41, 61.98, 56.28, 40.19, 38.47, 34.57, 33.86, 28.67, 24.60, 18.25.

Typical synthesis of P(DMAEMA) via RAFT polymerization. CPAETC (24.9 mg, 9.47×10^{-5} moles), DMAEMA (2.25 g, 1.43×10^{-2} moles), 1,4-dioxane (3.0 g), a 1% (w/w) ACVA in 1,4-dioxane (318,4 mg, 1.1×10^{-5} moles) and 1,3,5-trioxane (external NMR standard, 25 mg) were

introduced to a 8 mL microwave vial equipped with a magnetic stirring bar. The vial was sealed, and the solution deoxygenated by bubbling argon through it for 10 min. The vial was placed in an oil bath at 70 °C and allowed to stir for 7 h. The polymer was precipitated three times from THF into cold hexane and dried under reduced pressure to give a yellow solid.

Typical synthesis of P(DMAEMA-b-[nBMA-st-LAMA]) via RAFT polymerization. A portion of the precursor P(DMAEMA) macro-CTA (347.0 mg, 2.29×10^{-5} moles), *n*BMA (557.0 mg, 3.92×10^{-3} moles), LAMA (220.0 mg, 6.88×10^{-4} moles), THF (4.1 g), a 0.5% (w/w) solution of ACVA in THF (250 mg, 4.46×10^{-6} moles) and 1,3,5-trioxane (external NMR standard, 20.0 mg) were introduced to a 8 mL microwave vial equipped with a magnetic stirring bar. The vial was sealed, and the solution deoxygenated by bubbling argon through it for 10 min. The vial was placed in an oil bath at 70 °C and allowed to stir for 7 h. The polymer was precipitated three times from THF into cold hexane and dried under reduced pressure to give a yellow solid.

Synthesis of P(DMAEMA-b-[nBMA-st-HEMA]) via RAFT polymerization. A portion of the precursor *P(DMAEMA)* macro-CTA (200.0 mg, 1.41×10^{-5} moles), *n*BMA (339.9 mg, 2.39×10^{-3} moles), HEMA (54.9 mg, 4.22×10^{-4} moles), THF (3.68 g), a 1.0% (w/w) solution of ACVA in THF (105.0 mg, 3.75×10^{-6} moles) and 1,3,5-trioxane (external NMR standard, 31.0 mg) were introduced to a 8 mL microwave vial equipped with a magnetic stirring bar. The vial was sealed, and the solution deoxygenated by bubbling argon through it for 10 min. The vial was placed in an oil bath set at 70 °C and allowed to stir for 7 h. The polymer was precipitated three times from THF into cold hexane and dried under reduced pressure to give a pale yellow solid.

Synthesis of P(DMAEMA-b-nBMA) via RAFT polymerization. A portion of the precursor *P(DMAEMA)* macro-CTA (200.0 mg, 1.41×10^{-5} moles), *n*BMA (339.8 mg, 2.39×10^{-3} moles),

THF (3.52 g), a 1.0% (w/w) solution of ACVA in THF (94.0 mg, 3.35×10^{-6} moles) and 1,3,5-trioxane (external NMR standard, 28.0 mg) were introduced to a 8 mL microwave vial equipped with a magnetic stirring bar. The vial was sealed, and the solution deoxygenated by bubbling argon through it for 10 min. The vial was placed in an oil bath set at 70 °C and allowed to stir for 7 h. The polymer was precipitated three times from THF into cold hexane and dried under reduced pressure to give a pale yellow solid.

Calculations for RAFT Polymerization.

Monomer conversion (p) was calculated from ^{1}H NMR data by comparing the integrals of vinyl peaks (5.5-6.3 ppm) against an external reference (1,3,5-trioxane, 5.14 ppm) before (t = 0) and after (t = final) polymerization. The theoretical number-average molar mass ($M_{n,th}$) was then calculated using equation 3a and b for PDMAEMA and P(DMAEMA-*b*-[*n*BMA-*st*-LAMA]) respectively:

$$M_{n,th}\left(\frac{g}{mol}\right) = (Mw_{DMAEMA} * DP * p) + Mw_{CTA} \quad \ldots\ldots \text{(3a)}$$

$$M_{n,th}\left(\frac{g}{mol}\right) = (Mw_{LAMA} * DP * p) + (Mw_{nBMA} * DP * p) + Mw_{macroCTA} \quad \ldots\ldots \text{(3b)}$$

Where DP is the target degree of polymerization of each monomer and, Mw_{DMAEMA}, $Mw_{n\mathrm{BMA}}$, Mw_{MMA}, Mw_{CTA} and Mw_{macroCTA} are the molecular weight of the monomers, CTA and macro-CTA (PDMAEMA), respectively, and p is the monomer conversion of each monomer. Note: same formula was used for control polymers by substituting variables.

Table S1. Amount of different substances used for polymerization of polymers.

	PDMAEMA	P(DMAEMA-*b*-[*n*BMA-*st*-LAMA])	PDMAEMA	P(DMAEMA-*b*-*n*BMA)	P(DMAEMA-*b*-[*n*BMA-*st*-LAMA])	P(DMAEMA-*b*-[*n*BMA-*st*-HEMA])
Monomer	DMAEMA	LAMA, *n*BMA	DMAEMA	*n*BMA	LAMA, *n*BMA	HEMA, *n*BMA
$DP_{n,\text{target}}$	150	200	150	170	200	200
m_{CTA} added (mg)	50	250.0	50	200	200	200
n_{CTA} added (moles)	1.90×10^{-4}	1.65×10^{-5}	1.9×10^{-4}	1.44×10^{-5}	1.14×10^{-5}	1.14×10^{-5}
$m_{monomer}$ added (mg)	4483	557.7	4483	340	474.2	394.8
$n_{monomer}$ added (moles)	2.85×10^{-2}	3.31×10^{-3}	2.85×10^{-2}	2.39×10^{-3}	2.81×10^{-3}	2.81×10^{-3}
m_{ACVA} added (mg)	4.26	1.24	4.26	0.89	1.05	1.05
n_{ACVA} added (moles)	1.5×10^{-5}	4.4×10^{-6}	1.5×10^{-5}	3.19×10^{-6}	1.41×10^{-6}	3.75×10^{-6}
Dioxane added (µL)	2361	4583	2470	3500	4060	4140
CPAETC/ACVA	12.5	3.8	12.5	4.41	3.75	13.7
T (°C)	70	70	70	70	70	70
Time (min)	420	420	420	420	420	420

From left to right, each PDMAEMA was a precursor of the subsequent block copolymer/s.

Critical Micelle Concentration.

Critical micelle concentrations (CMC) were determined by fluorescence measurements at 25 °C with a Tecan Infinite M200 PRO microplate reader, using Nile Red as the fluorescence dye. A bulk solution of each polymer with concentrations ranging from 3.8-4.6 mg mL^{-1} were prepared and a solution of Nile Red in THF, 3.14×10^{-4} M (0.01 mg mL^{-1}). Sample concentrations ranging from 1.0 mg mL^{-1} to 0.001 mg mL^{-1} of polymer were prepared in a vial by diluting the stock solutions, followed by a solution of Nile Red (0.01 mg mL^{-1}) and incubated for 4 h at room temperature under reduced pressure. Then 100 µL of each sample was transferred to a 96-well plate and equilibrated at each temperature for 30 min. The fluorescence was measured in 96-well plates using an excitation wavelength of 535 nm. The fluorescence emission spectra were measured from 400 to 600 nm in 2 nm steps. For CMC determination the maximum of each fluorescence emission spectra was plotted versus the micelle concentration for each sample,

respectively. The CMC was determined as the intersection point in the plot of the maximum fluorescence emission versus the micelle concentration.

Dynamic Light Scattering (DLS) Measurements.

The hydrodynamic diameters of the nanoassemblies were monitored by DLS using a Zetasizer Nano ZS (Malvern Instruments, Germany) with a He–Ne laser operating at a wavelength of 633 nm. Each sample was measured in triplicates at 25 °C with measurement duration set to automatic (about 10 min) after an equilibration time of 120 s. The counts were detected at an angle of 173°. The mean particle size was approximated as the effective (z-average) diameter and the width of the distribution as the polydispersity index of the particles (PDI) obtained by the cumulants method assuming a spherical shape. Data are expressed as mean ± SD of three technical repetitions. Each micelle suspension was measured after filtration (0.20 µm polytetrafluoroethylene (PTFE) membrane) except for P($DMAEMA_{101}$-*b*-[*n*BMA_{124}-*st*-$LAMA_{22}$]). Filtered bulk polymer solutions were stored at room temperature (RT).

The size (hydrodynamic diameter) of the polyplexes was measured following polyplex preparation at N*/P 30 in 100 µL HBG buffer as described in the manuscript. Each sample was measured in triplicates with three runs of 30 s at 25 °C after an equilibration time of 30 s. Data are expressed as mean of three technical replicates.

N*/P Ratio Calculations.

The N*/P ratio was defined as the ratio of the total amount of protonatable amines in polymer solution in relation to the total amount of phosphates in the pDNA solution.

The volume of polymer needed to prepare polyplexes with 15 µg mL^{-1} pDNA at different N*/P ratios was calculated as described by the following equations:

$$V_{total} \cdot P = V_{poly} \cdot N_{poly}$$

$$V_{poly} = \frac{V_{total} \cdot P}{N_{poly}}$$

$$V_{poly} = V_{total} \cdot \frac{n_{pDNA} \cdot P}{n_{poly} \cdot N}$$

$$V_{poly} = V_{total} \cdot \frac{m_{pDNA} \cdot P \cdot M_{poly}}{m_{poly} \cdot N \cdot M_{pDNA}}$$

Where V_{total}, P, V_{poly} and N_{poly} are the total required volume, the total number of phosphates of the pDNA, the required volume of polymer and the total number of active amines of the polymer, respectively.

Heparin dissociation assay.

Table S2. Kinetic cycle protocol for automated heparin addition by the microplate reader

**Kinetic cycle**	**Repetitions**	***Addition of heparin***		***Orbital shake***	***Incubation***	***Measurement***
		V / µL	Stock Solution / U mL^{-1}			
1	2	5	100	10 s	10 min, 37°C	λ_{Ex} = 525 nm / λ_{Em} = 605 nm
2	1	15	100	10 s	10 min, 37°C	λ_{Ex} = 525 nm / λ_{Em} = 605 nm
3	3	5	500	10 s	10 min, 37°C	λ_{Ex} = 525 nm / λ_{Em} = 605 nm
4	1	10	500	10 s	10 min, 37°C	λ_{Ex} = 525 nm / λ_{Em} = 605 nm

The heparin concentration needed to release 70% of pDNA was calculated with OriginPro, Version 2018b (OriginLab Corporation, US) using a logistic function fitted to the respective single measurement points (n = 3) of each polymer (4).

$$y = \frac{A_1 - A_2}{1+(x/x_0)^p} + A_2 \quad (4)$$

Where A_1, A_2, x_0 and p are the initial value, the final value, the center and the power of the curve, respectively. The HC_{70}-values (y = 70) were calculated by substitution of the respective values into the equation.

Gel Retardation Assay (GRA).

The complex formation of the polymers with pDNA was further investigated with the GRA. Briefly, polyplexes were formed at varying N*/P ratios as described in the respective section. Following the 15 min incubation, the samples were diluted 1:6 with Green Gel Loading Buffer and run on a 1% (w/v) agarose gel containing 0.1 µg mL^{-1} ethidium bromide (EtBr) at 80 V for 1 h. The gel was images using the Red™ Imaging System (Alpha Innotech, Kasendorf, Germany).

Erythrocyte Aggregation and Hemolysis.

The interaction of polymers with cellular membranes was examined by analyzing the release of hemoglobin from erythrocytes as published before.[3, 4] Blood from human donors, collected in tubes with citrate, was obtained from the Department of Transfusion Medicine of the University Hospital, Jena. The blood was centrifuged without pooling at 4,500 × g for 5 min, and the pellet was washed three times with cold phosphate buffered saline (PBS, pH 7.4). Following a 10 times dilution with PBS (either pH 7.4 or pH 6.0), 500 µL aliquots of erythrocyte suspension were mixed 1:1 with the polymer solutions, which were prepared with PBS pH 7.4 or pH 6.0, and incubated at 37 °C for 60 min. After centrifugation at 2,400 × g for 5 min, the supernatant was transferred to a clear flat bottomed 96-well plate (VWR, Germany) and the hemoglobin release was determined as the hemoglobin absorption at $\lambda = 544$ nm. Absorption at $\lambda = 630$ nm was used as reference. Complete hemolysis (100 %) was achieved using 1 % Triton X-100 as positive control. Pure PBS was used as negative control (0 % hemolysis). The hemolytic activity of the polycations was calculated as follows (5):

$$\text{Hemolysis / \%} = \frac{(A_{Sample} - A_{Negative\ control})}{(A_{Positive\ control} - A_{Negative\ control})} \cdot 100 \qquad (5)$$

Where A_{Sample}, $A_{Negative\ control}$ and $A_{Positive\ control}$ are the absorption values of a given sample, the PBS treatment and the Triton X-100 treatment, respectively. A value less than 2% hemolysis rate was classified as non-hemolytic, 2 to 5% as slightly hemolytic and values > 5% as hemolytic.

To determine the cell aggregation, erythrocytes were isolated as described above. Subsequently, 100 µL of the erythrocyte-polymer suspension were transferred to a clear flat bottomed 96-well plate (VWR, Germany). The cells were incubated at 37 °C for 2 h, and the absorbance was measured at λ = 645 nm. Cells treated with PBS served as negative control and cells treated with 50 µg mL^{-1} 10 kDa bPEI were used as positive control. Aggregation potential of the polymers was calculated as follows (6):

$$\text{Aggregation} = \frac{A_{Negative\ control}}{A_{Sample}} \qquad (6)$$

Where A_{Sample} and $A_{Negative\ control}$ are the absorption values of a given sample and the PBS treatment, respectively. Experiments were run in technical triplicates and were performed with blood from three different blood donors.

Polyplex Uptake with CLSM and Image Processing.

For uptake studies *via* confocal laser scanning microscopy (CLSM), cells were seeded and treated as described for flow cytometry analysis, but in glass-bottomed dishes (CellView cell culture dishes with four compartments, Greiner Bio-One) and analyzed following incubation with polyplexes containing YOYO-1 labeled pDNA (0.027 nmol per 1 µg pDNA) and indicated polymers for 1 h. To image intracellular distribution in living cells, Hoechst 33342 was added for 5 min to stain cell nuclei. To stain acidic compartments inside the cells, 50 nM LysoTracker™ Red (LTR) were added for 5 min. Prior to imaging, trypan blue was added to a final concentration of 0.04% to quench fluorescence of YOYO-1 outside the cells. Live cell imaging was performed

using a LSM880, Elyra PS.1 system (Zeiss, Germany) applying the argon laser for excitation at 488 nm (2%) and 405 nm (0.5%), emission filters for 410-479 nm (Hoechst) and 508-553 nm (YOYO-1) with a gain of 700, respectively. LTR was excited at 561 nm and detected with an emission filter for 589-690 nm and a gain of 650. To avoid cross talk between the different channels, Hoechst and LTR were imaged simultaneously in one track and YOYO-1 in a second track. For fast imaging, the tracks were switched in every line of the image. The images were acquired as z-stacks of 6 slices around the center of the cells (in total 7 µm for HEK293T and 15 µm for K-562 cells) to image also polyplexes not present in the center of the cells. For magnification, a 40 × 1.4 NA plan apochromat oil objective was applied. Images were acquired using the ZEN software, version 2.3 SP1 (Zeiss, Germany). The experiments were performed at least twice. All images were processed in batch mode using ImageJ, version 1.52.[4] At first, the 3D data of the images were turned into 2D images *via* maximum intensity projection of all 6 slices. Subsequently, the lower and upper limits of the grey values were adjusted for each channel to enhance the contrast of the images. Same values were applied to all images within one cell line.

FURTHER RESULTS

Characterization of Polymers.

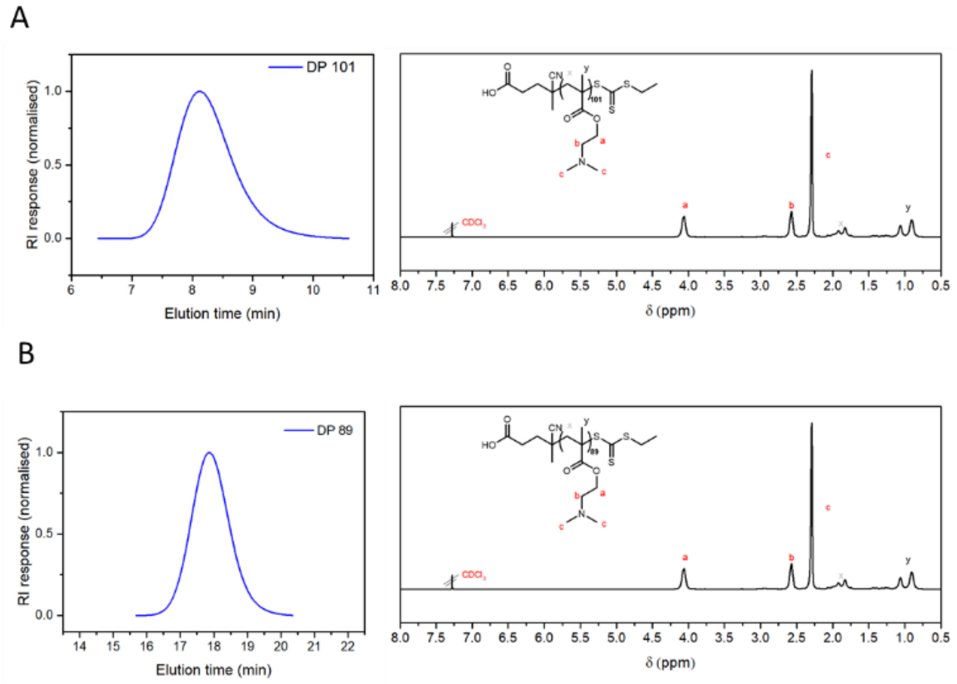

Figure S1. Characterization of $PDMAEMA_{101}$ and $PDMAEMA_{89}$.

Conversion from ^{1}H NMR, molar masses determined by (A) ($CHCl_3$/IPA/NEt_3) SEC traces– PMMA calibration and (B) (DMAc + 0.21 % LiCl) SEC traces– PMMA calibration.

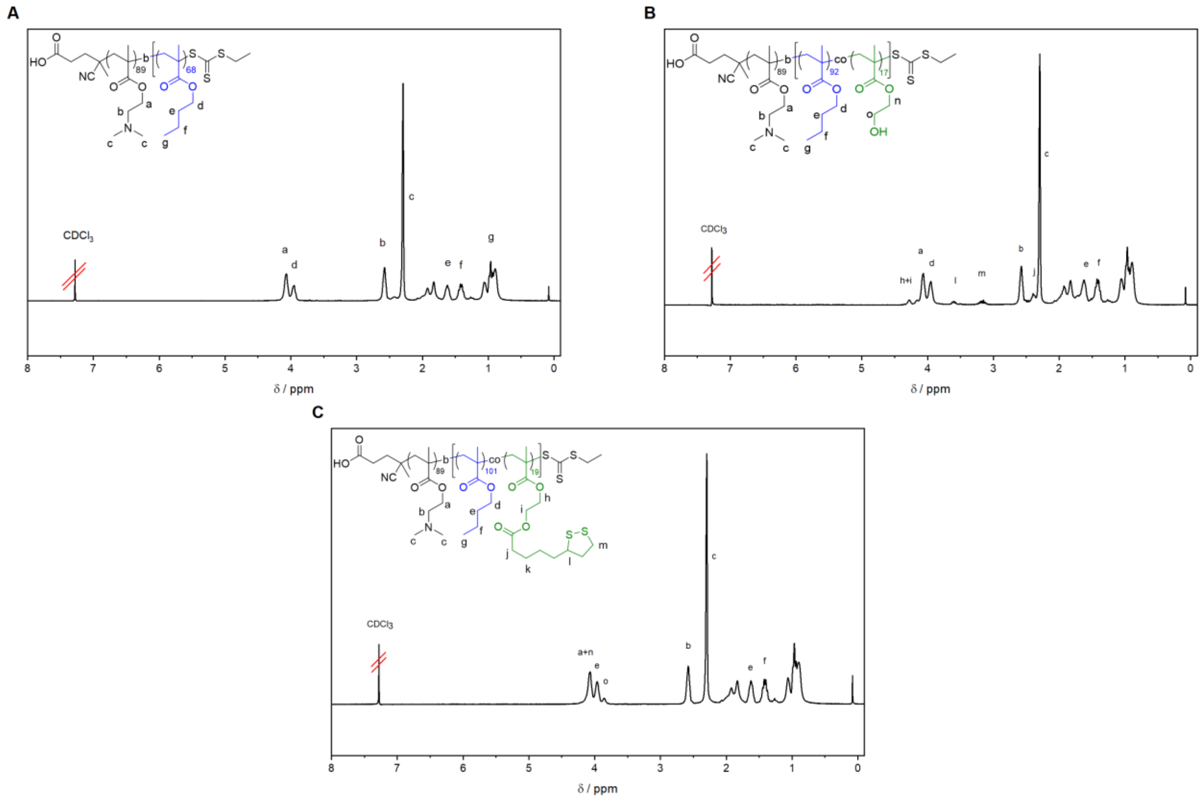

Figure S2. NMR results.

(A) P(DMAEMA$_{89}$-*b*-*n*BMA$_{68}$), **(B)** P(DMAEMA$_{89}$-*b*-[*n*BMA$_{92}$-*st*-HEMA$_{17}$]), **(C)** P(DMAEMA$_{89}$-*b*-[*n*BMA$_{101}$-*st*-LAMA$_{19}$]).

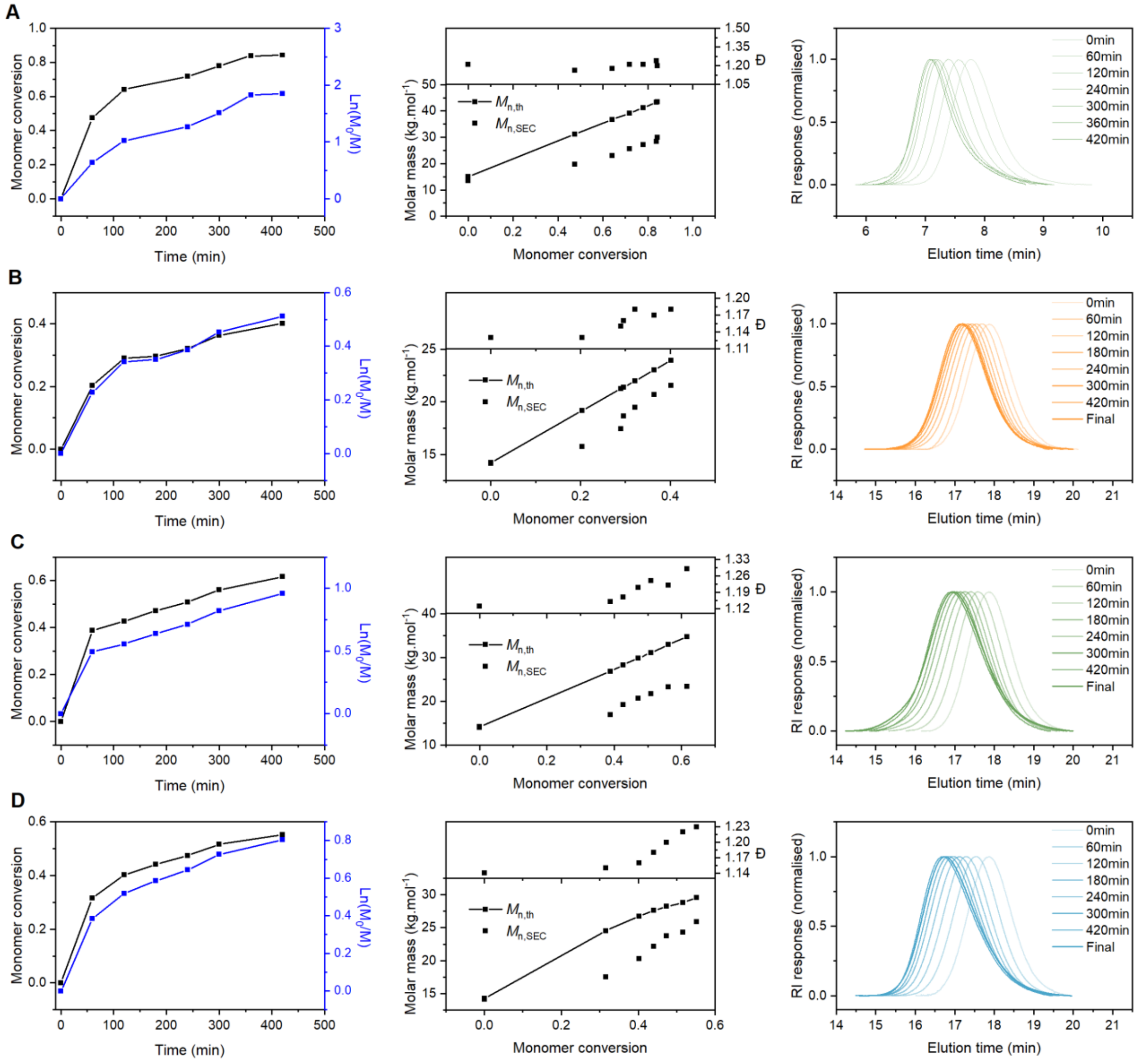

Figure S3. Kinetic results.

(A) P(DMAEMA$_{101}$-*b*-[*n*BMA$_{120}$-*st*-LAMA$_{22}$]), **(B)** P(DMAEMA$_{89}$-*b*-*n*BMA$_{68}$) **(C)** P(DMAEMA$_{89}$-*b*-[*n*BMA$_{101}$-*st*-LAMA$_{19}$]), **(D)** P(DMAEMA$_{89}$-*b*-[*n*BMA$_{92}$-*st*-HEMA$_{17}$]). Conversion from ^{1}H NMR, molar masses determined by ($CHCl_3$/IPA/NEt_3) SEC traces-PMMA calibration (A) and SEC-9: DMAc + 0.21 % LiCl – PMMA calibration (B-D).

Table S3. Summary of polymer characterization.

Polymer-ID [a]	$M_{n,th}$ [b]	$M_{n,SEC}$ [c]	*Đ* [c]
	kg mol^{-1}		
$PDMAEMA_{101}$	16.1	13.8	1.17
P($DMAEMA_{101}$-*b*-[*n*BMA_{120}-*co*-$LAMA_{22}$])	39.2	30.0	1.19
$PDMAEMA_{89}$	14.2	14.0	1.15
P($DMAEMA_{89}$-*b*-*n*BMA_{68})	23.9	21.0	1.18
P($DMAEMA_{89}$-*b*-[*n*BMA_{92}-*co*-$HEMA_{17}$])	29.6	24.7	1.26
P($DMAEMA_{89}$-*b*-[*n*BMA_{101}-*co*-$LAMA_{19}$])	34.7	23.1	1.32

[a] Degree of Polymerization was determined *via* ^{1}H NMR.
[b] Determined using equation 6 of the ESI.
[c] Determined *via* $CHCl_3$-SEC and DMAc-SEC with PMMA standards.

Table S4. Summary of micelle characterization.

Code	Micelle					Polyplex			
	Stock-solution		370-540 µg mL^{-1}			370-540 µg mL^{-1}		37-54 µg mL^{-1}	
	Size [a]	PDI [b]	Size [a]	PDI [b]	CMC [c]	Size [a]	PDI [b]	Size [a]	PDI [b]
	nm		nm		µg mL^{-1}	nm		nm	
LAMA-mic	46.6	0.19	53.4	0.15	-	-	-	-	-
BMA-mic	19.5	0.25	46.6	0.10	26	56.8	0.21	73.4	0.38
HEMA-mic	21.7	0.19	51.3	0.08	30	54.1	0.10	63.3	0.26
LAMA-mic II	24.2	0.23	54.2	0.12	29	61.1	0.14	62.4	0.26

[a] Determination of the size as the hydrodynamic diameter *via* DLS (concentrations see Table S3).
[b] Determined *via* DLS (concentrations see Table S3).
[c] Determined using Nile Red encapsulation as fluorescence probe.

DLS Measurement of Micelles.

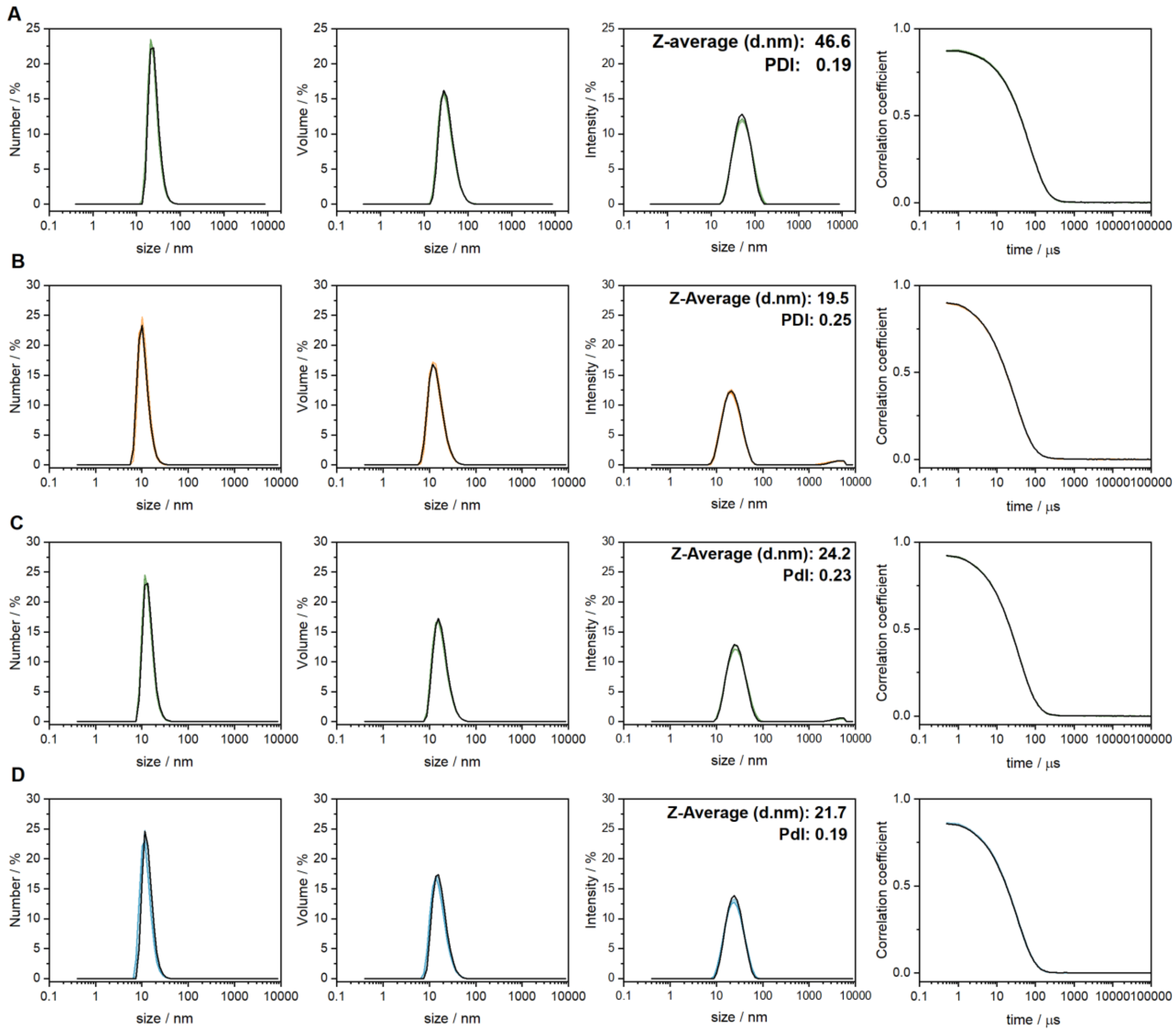

Figure S4. DLS measurements of the micelles.

(A) LAMA-mic, (B) BMA-mic, (C) LAMA-mic II and (D) HEMA-mic; stock solutions.

Original Cryo-TEM images

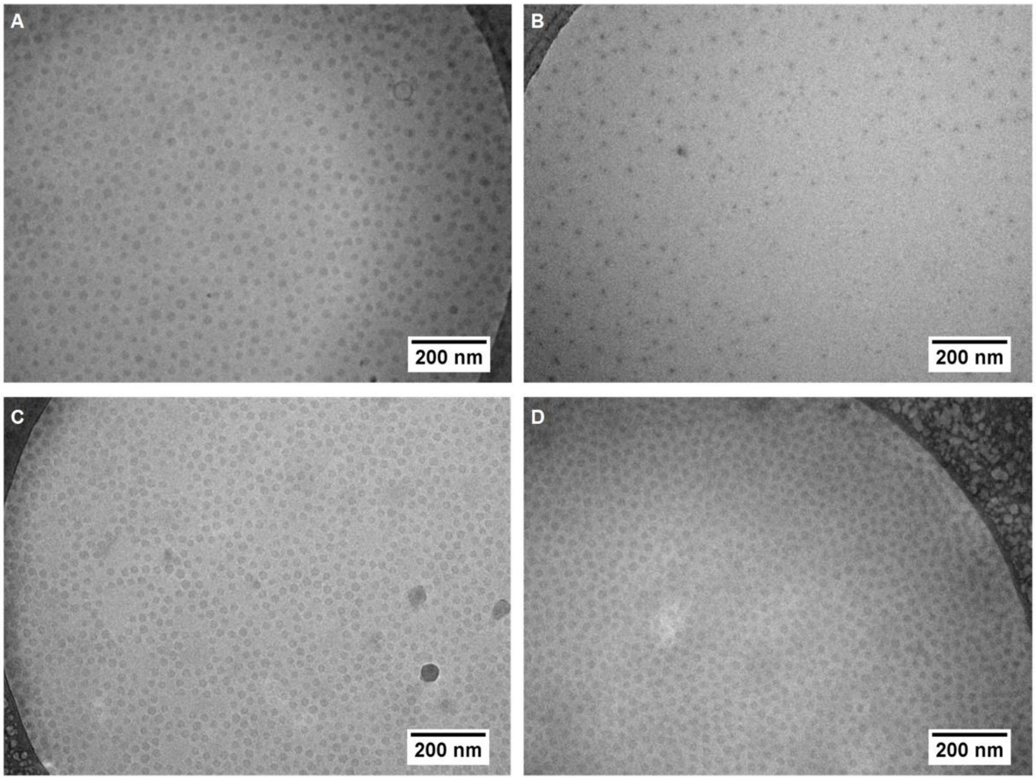

Figure S5. Original cryo-TEM images of micelles.

(A) LAMA-mic, **(B)** BMA-mic, **(C)** LAMA-mic II, **(D)** HEMA-mic.

Further Biological Results.

Table S5. Polymer concentrations in different assays.

	Stock solutions	EBA/HRA, DLS		Cell—based assays		Amount of lipoic acid on cells	
	µg mL^{-1}	µg mL^{-1}		µg mL^{-1}		µM	
	-	N*/P 15	N*/P 30	N*/P 15	N*/P 30	N*/P 15	N*/P 30
pDNA	-	15	15	1.5	1.5	-	-
LPEI	1000	30	59	3.0	5.9	-	-
PDMAEMA	10000	111	221	11.1	22.1	-	-
LAMA-mic	2100	275	549	27.5	54.9	14.8	29.5
PDMAEMA II	10000	111	221	11.1	22.1	-	-
BMA-mic	4940	189	371	18.9	37.1	-	-
HEMA-mic	4600	228	455	22.8	45.5	-	-
LAMA-mic II	4580	269	538	26.9	53.8	14.8	29.5

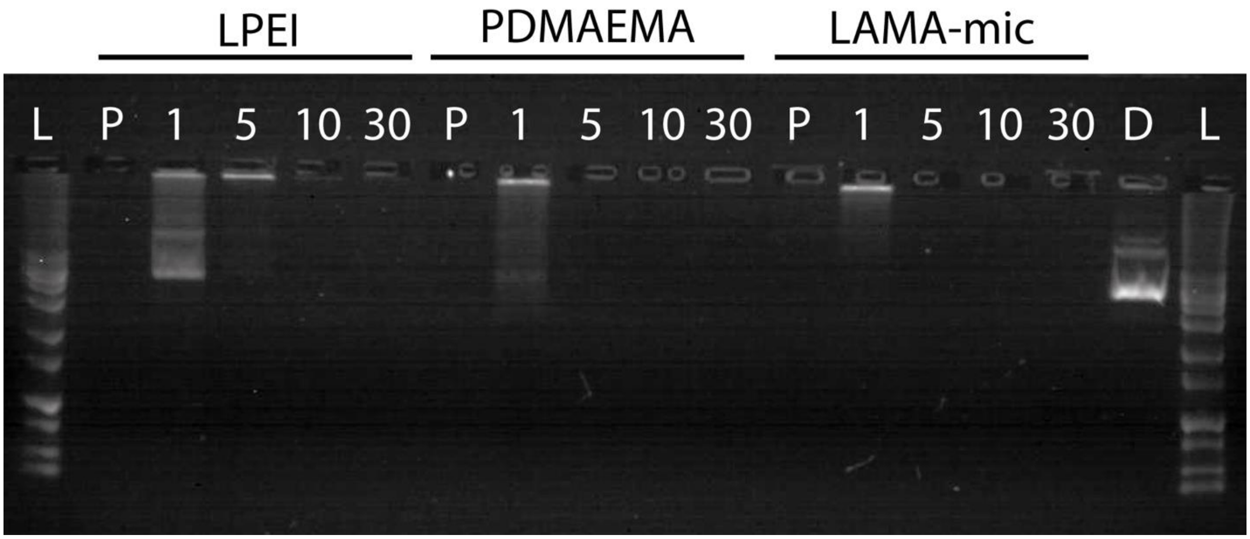

Figure S6. Gel retardation assay.

The agarose gel was loaded with polyplexes prepared as described before. L: High Range DNA Ladder, P: only Polymer at concentrations equal to N*/P 30, numbers indicate the respective N*/P ratios, D: pDNA in HBG buffer at the same concentration used for polyplexes.

Table S6. Computational analysis of monomer hydrophobicity by Molinspiration.

	EI	DMAEMA	*n*BMA	LAMA
Structure	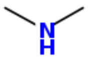	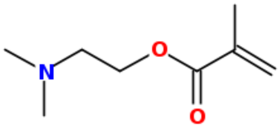	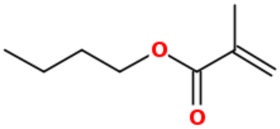	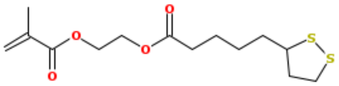
MW	45.09	157.21	142.20	318.46
logP[1]	0.1	1.4	2.81	3.43

[1]The logP values were calculated using the Molinspiration Property Calculation Service of the Molinspiration Cheminformatics website (https://www.molinspiration.com/cgi-bin/properties).

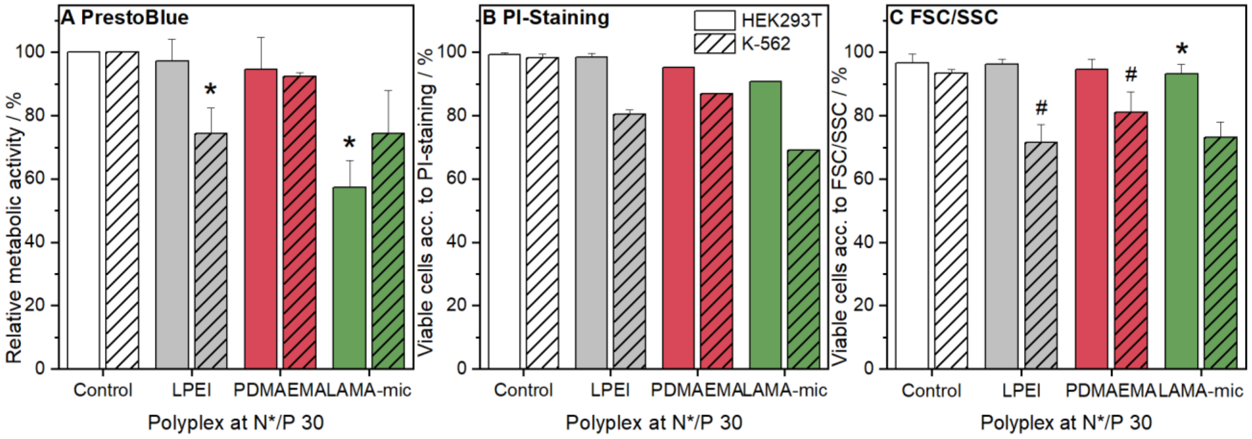

Figure S7. Cytotoxicity of polymers in HEK293T and K-562 cells.

(A) Metabolic activity using the PrestoBlue™ assay following incubation of cells with polyplexes of indicated polymers and pDNA at N*/P 30 for 24 h. Values represent mean ± SD of n = 3. (B) Membrane integrity using propidium iodide (PI) staining and flow cytometry following incubation of cells with polyplexes of indicated polymers and pDNA at N*/P 30 for 24 h. Viability was calculated as the difference of 100 and the percentage of PI positive cells gated in the ECD-channel. Values represent mean ± SD of n > 1. (C) Viable cells according to appearance of cells in FSC/SSC plot of flow cytometry. Values represent mean ± SD of n > 1.

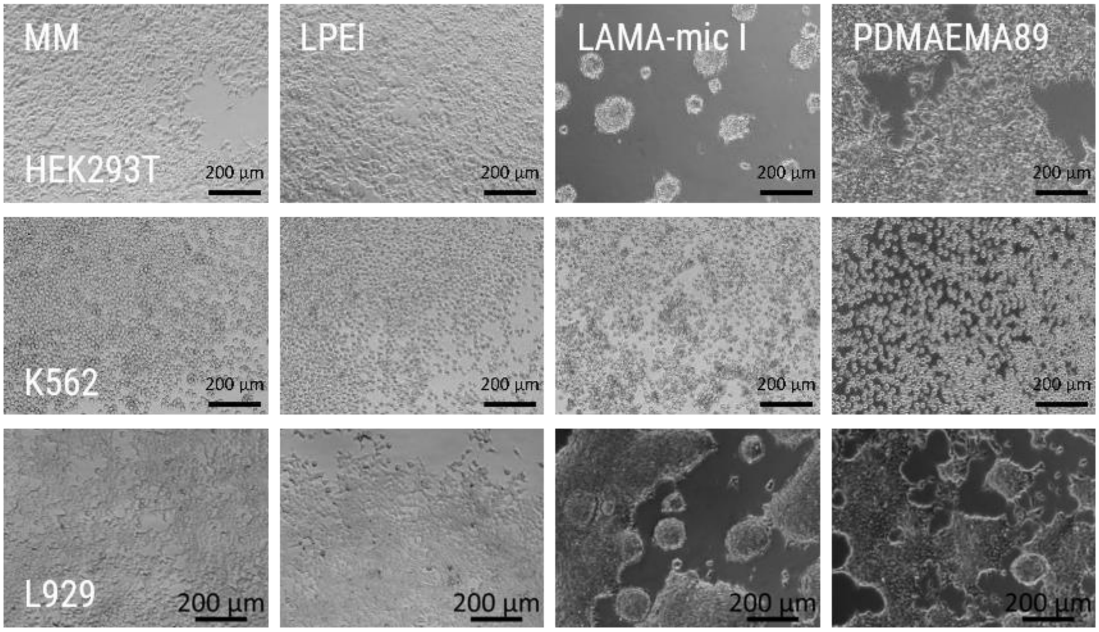

Figure S8. Spheroid formation in different cell lines.

Cells were incubated with polyplexes and pDNA at N*/P 30 for 24 h. Images were acquired *via* light microscopy.

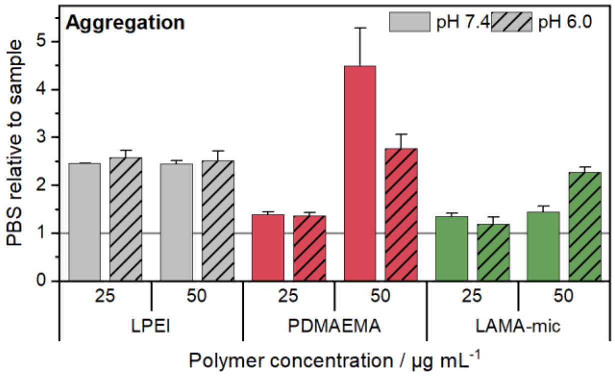

Figure S9. Interaction of polymers with erythrocyte membranes.

Human erythrocytes were washed and incubated with polymers at different concentrations in PBS of different pH values present in blood/cytoplasm (pH 7.4) or endosomal compartments (pH 6). Aggregation of indicated polymers was measured as light absorption by erythrocytes. Values are calculated as the negative control (PBS value) relative to the sample value and represent mean ± SD (n = 3).

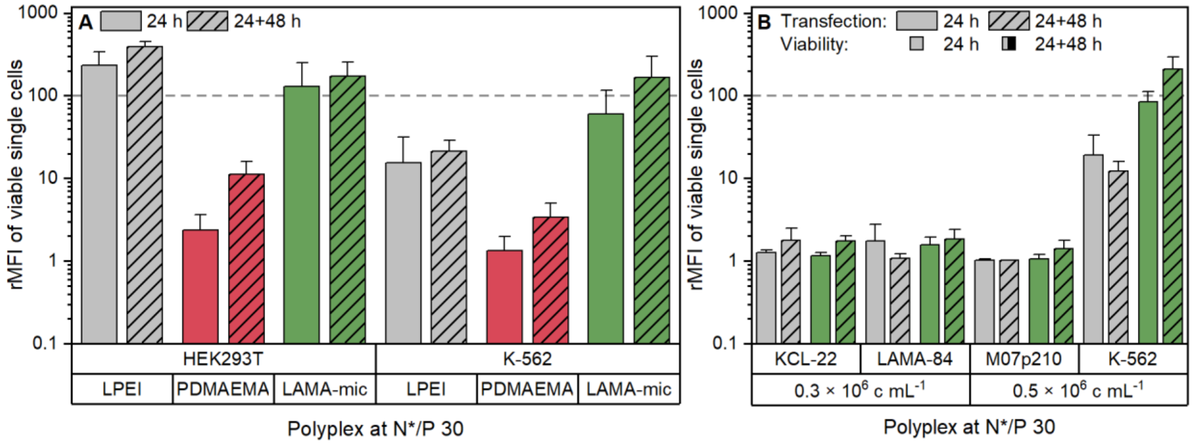

Figure S10. Transfection efficiency of LAMA-mic in different cell lines.

EGFP expression of viable cells was analyzed *via* flow cytometry following incubation of cells with polyplexes of mEGFP-N1 pDNA and polymers at N*/P 30 in respective growth medium (D10 or R10 with 10 mM HEPES) either for 24 h or for 24 h, splitting the cell suspension 1:2 and further incubation for 48 h. Values represent mean ± SD of rMFI values of viable, single cells (n ≥ 3).

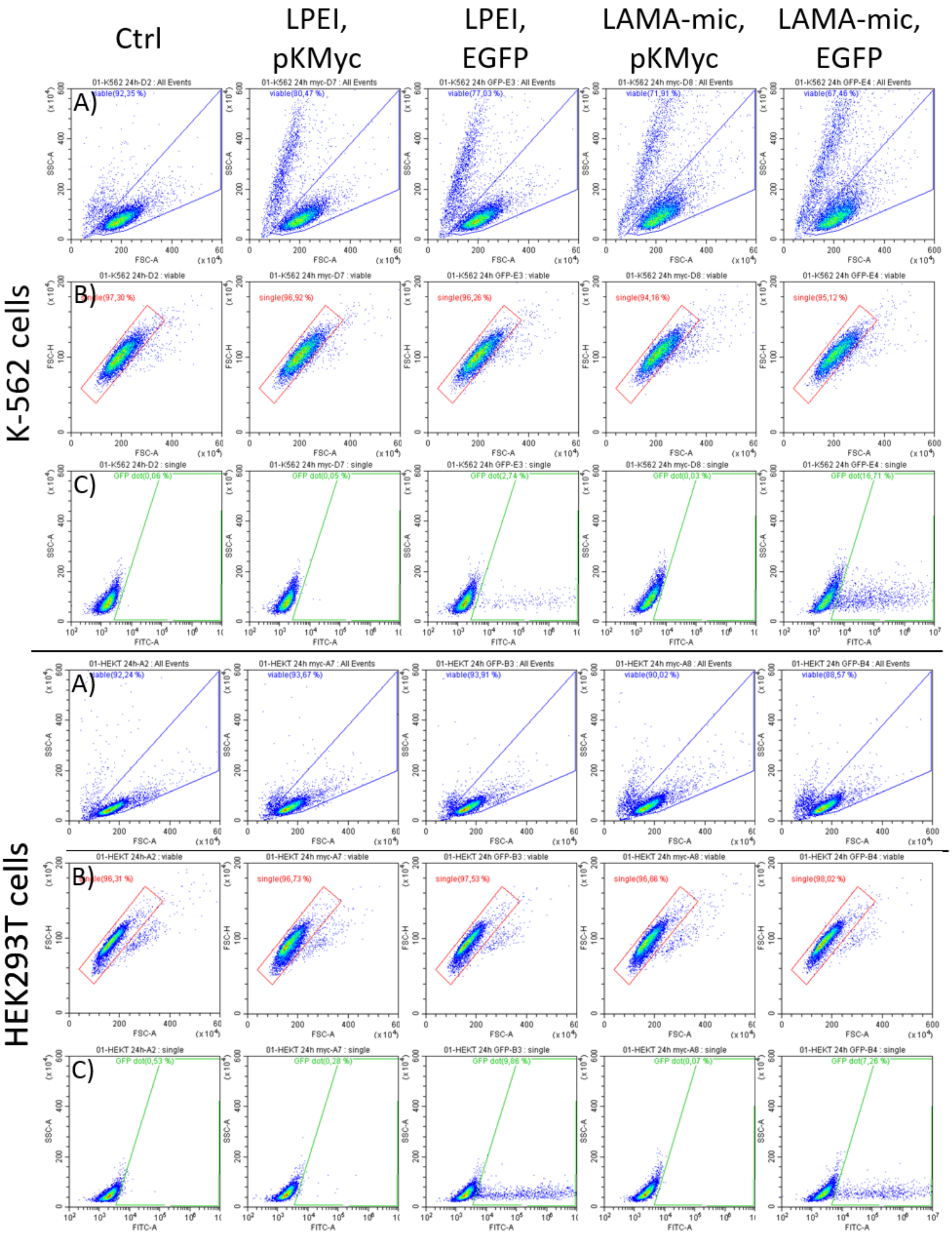

Figure S11. Gating strategy for pDNA transfection using the example of 24 h incubation. Viable single cells were gated in FSC/SSC and FSC-A/FSC-H dot plots (A,B). Subsequently cells with EGFP fluorescence were discriminated by gating to the respective pKMyc control.

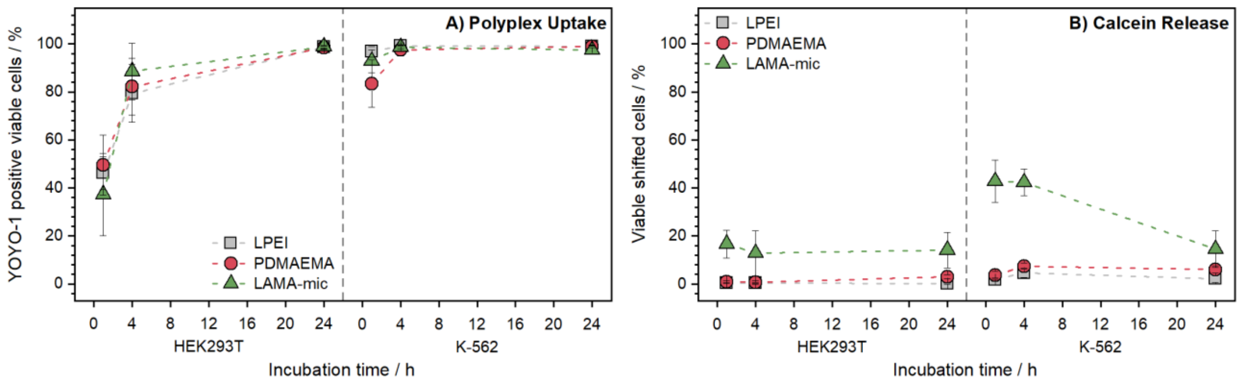

Figure S12. Investigation of the gene delivery process.

(A) Cellular internalization of LAMA-mic polyplexes in different cell lines. Cells were incubated with polyplexes of polymers and YOYO-1-labeled pDNA at N*/P 30 and analyzed *via* flow cytometry. Cells incubated with labeled pDNA served as control. Values represent mean ± SD of % cells showing YOYO-1 fluorescence higher than the control (n = 3). (B) Endosomal escape of LAMA-mic polyplexes detected by the non-permeable dye calcein and analyzed *via* flow cytometry. Values represent mean ± SD of % cells showing higher fluorescence intensity than the calcein control (n = 3).

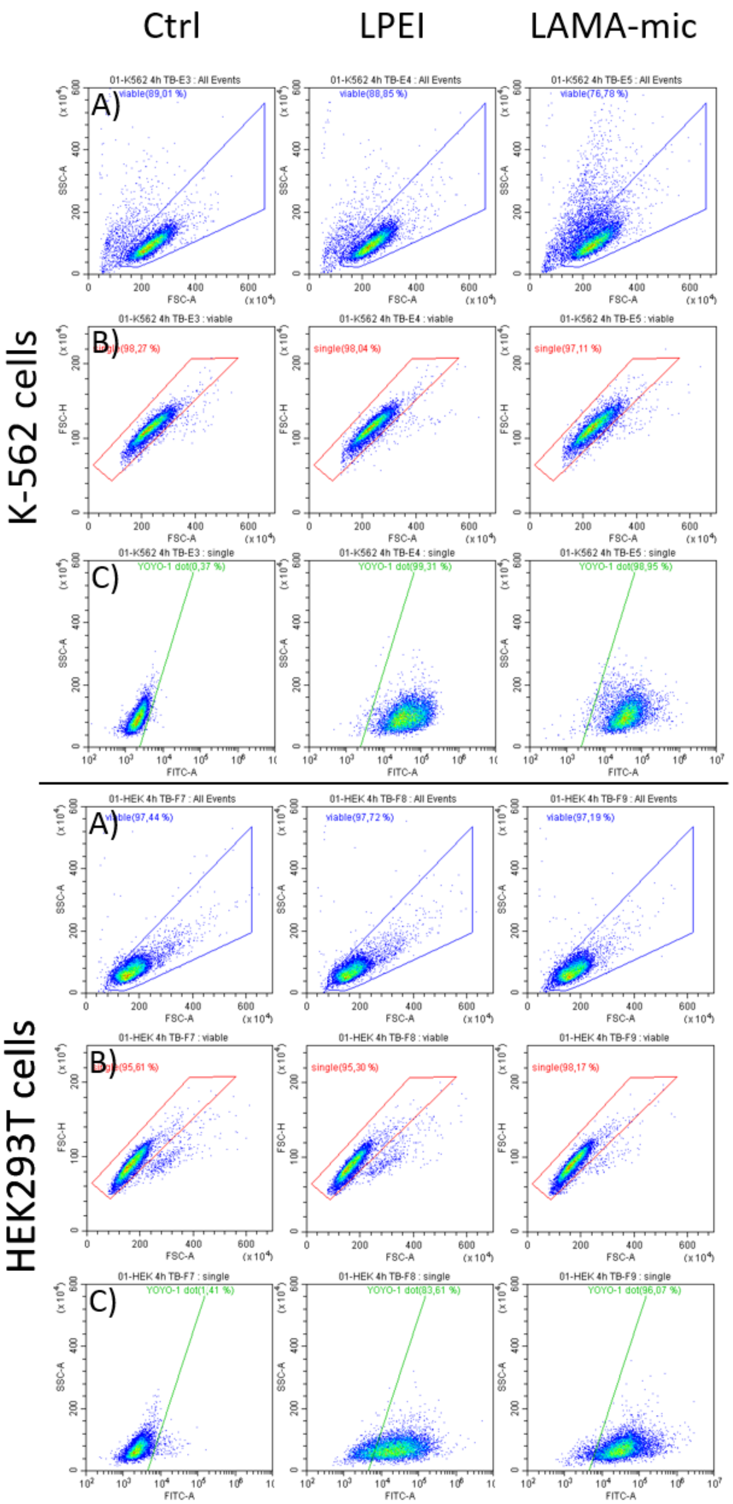

Figure S13. Gating strategy for polyplex uptake using the example of 4 h incubation.

Viable single cells were gated in FSC/SSC and FSC-A/FSC-H dot plots (A,B). Subsequently cells with YOYO-1 fluorescence were discriminated by gating to the pDNA-YOYO-1 control of the respective incubation time.

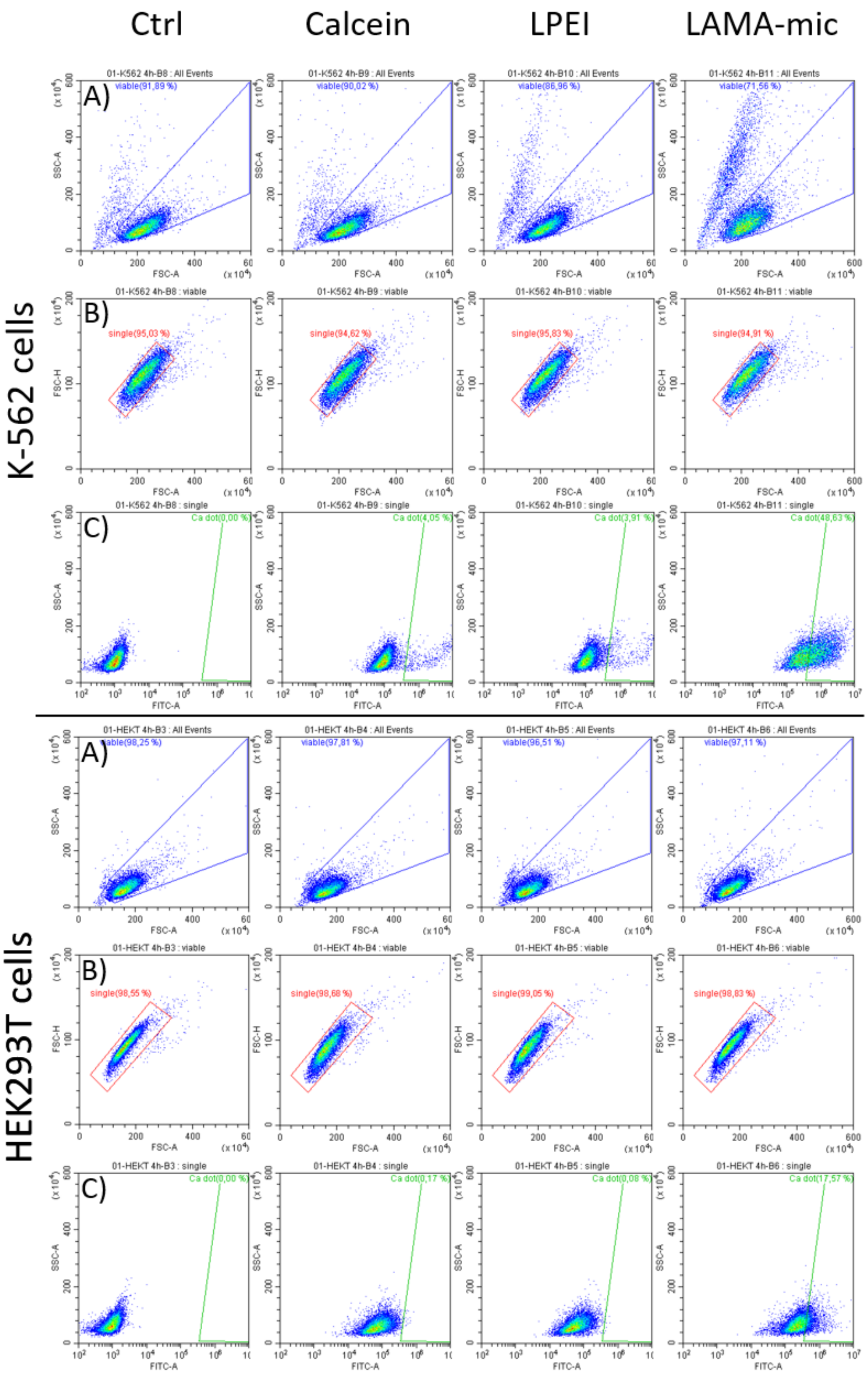

Figure S14. Gating strategy for calcein release using the example of the 4 h incubation. Viable single cells were gated in FSC/SSC and FSC-A/FSC-H dot plots (A,B). Subsequently cells with calcein fluorescence higher than the control were discriminated by gating to the calcein control of the respective incubation time.

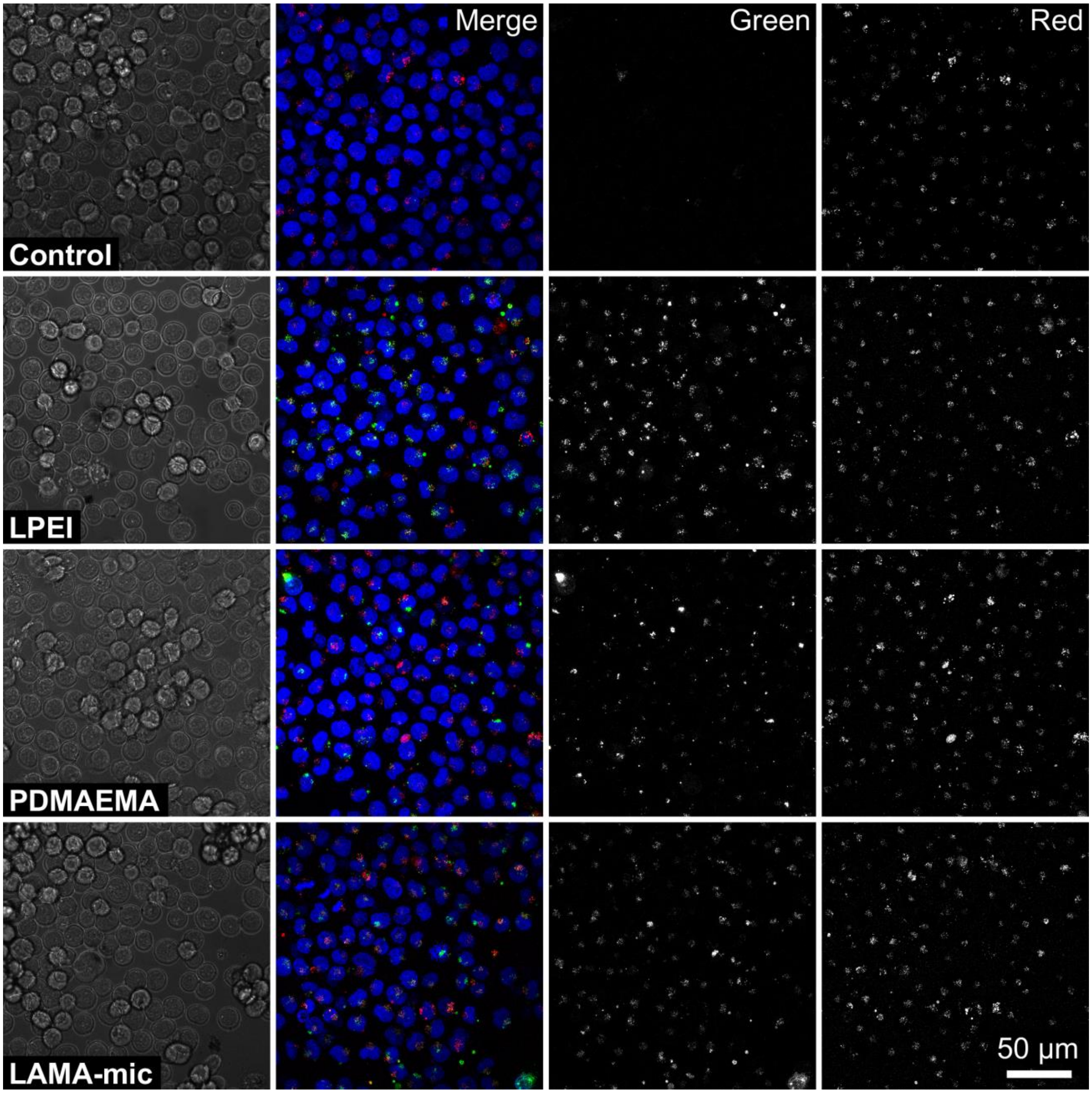

Figure S15. CLSM study of polyplex uptake in K-562 cells.

K-562 cells were incubated with polyplexes of YOYO-1-labeled pDNA and polymers at N*/P 30 in growth medium (R10 with 10 mM HEPES) for 1 h. Endolysosomes were stained with LysoTracker^{TM} Red (red), Nuclei were stained with Hoechst 33342 (blue) and YOYO-1 fluorescence (green) was quenched with trypan blue.

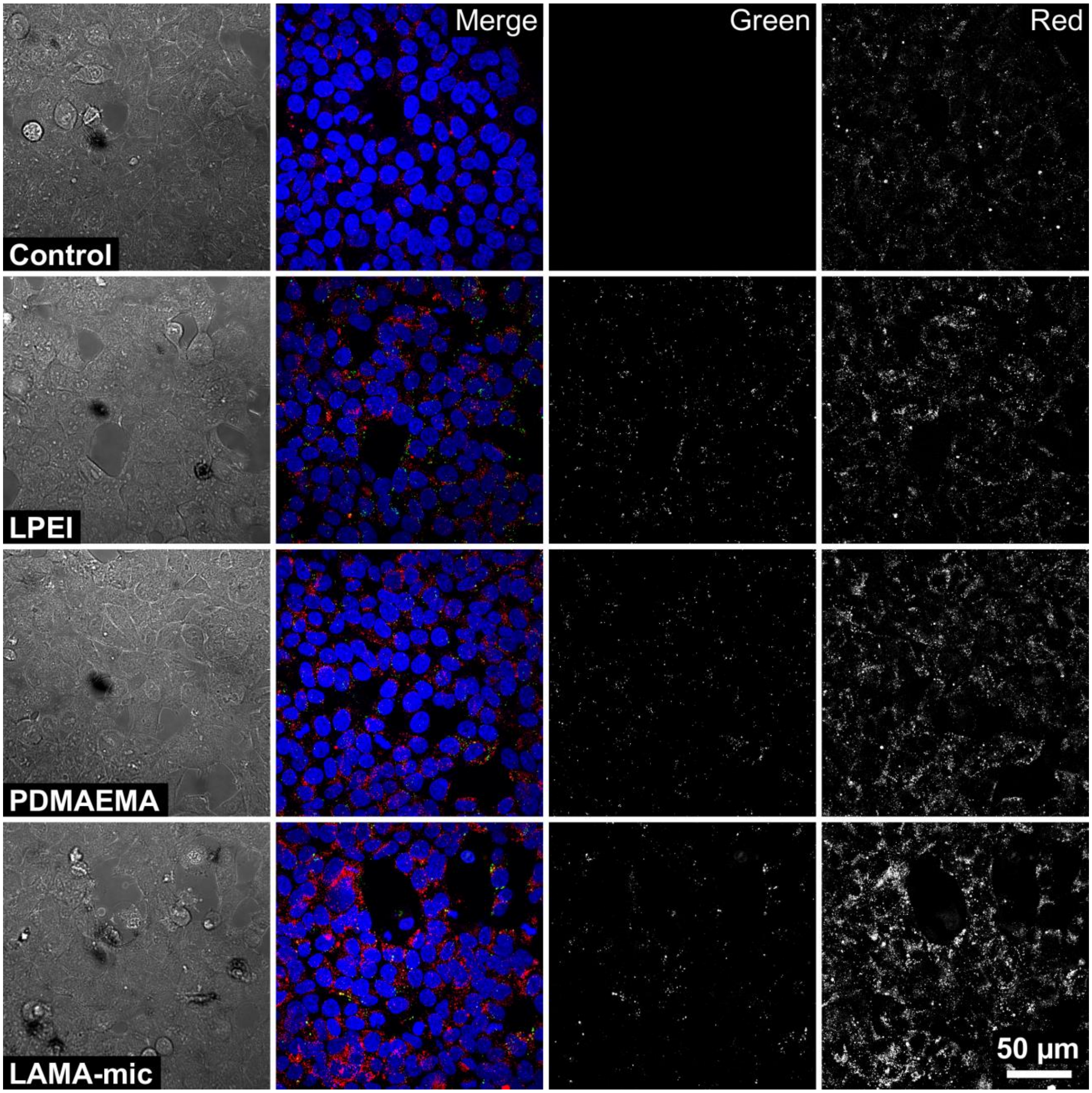

Figure S16. CLSM study of polyplex uptake in HEKT293T cells.

HEK293T cells were incubated with polyplexes of YOYO-1-labeled pDNA and polymers at N*/P 30 in growth medium (D10 with 10 mM HEPES) for 1 h. Endolysosomes were stained with LysoTracker^{TM} Red (red), nuclei were stained with Hoechst 33342 (blue) and YOYO-1 fluorescence (green) was quenched with trypan blue.

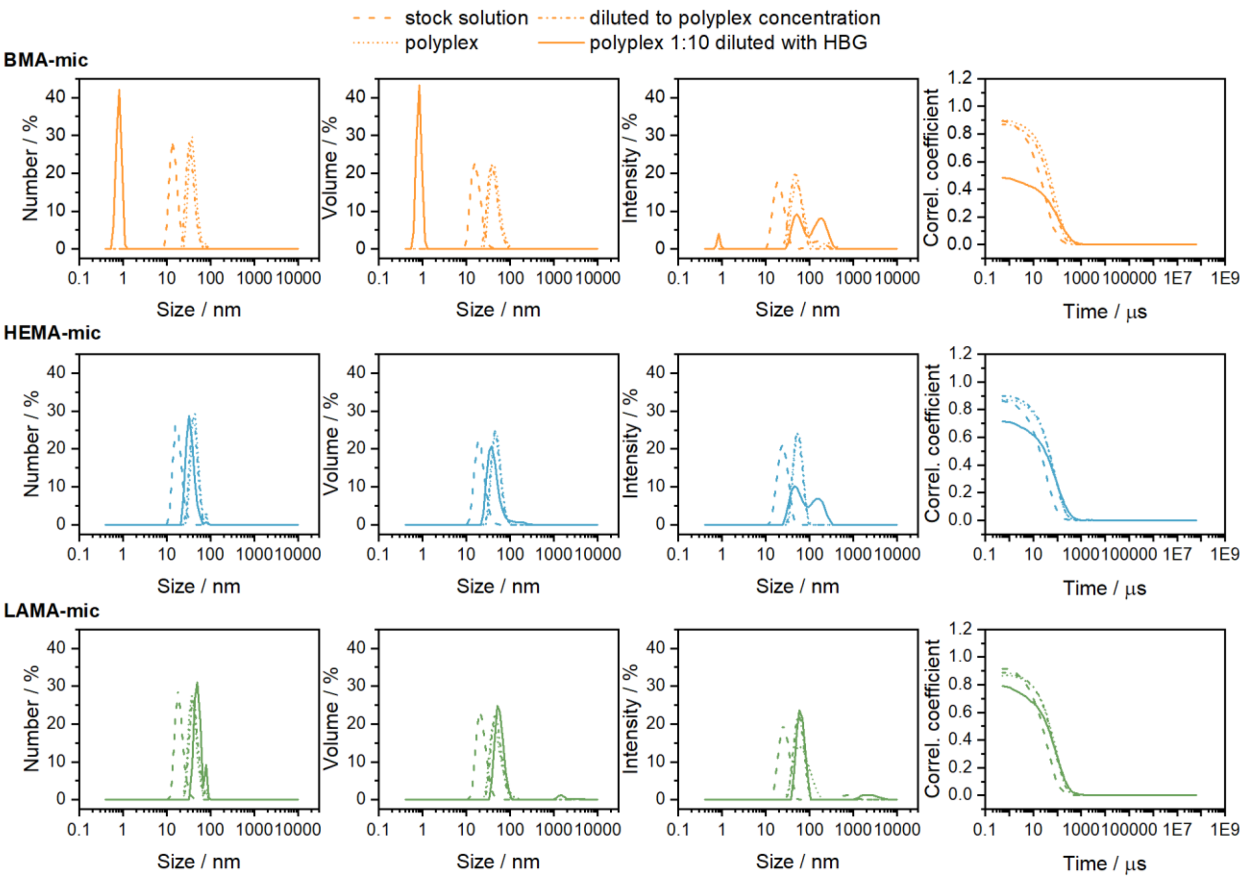

Figure S17. DLS measurement of the micelles at different concentrations.

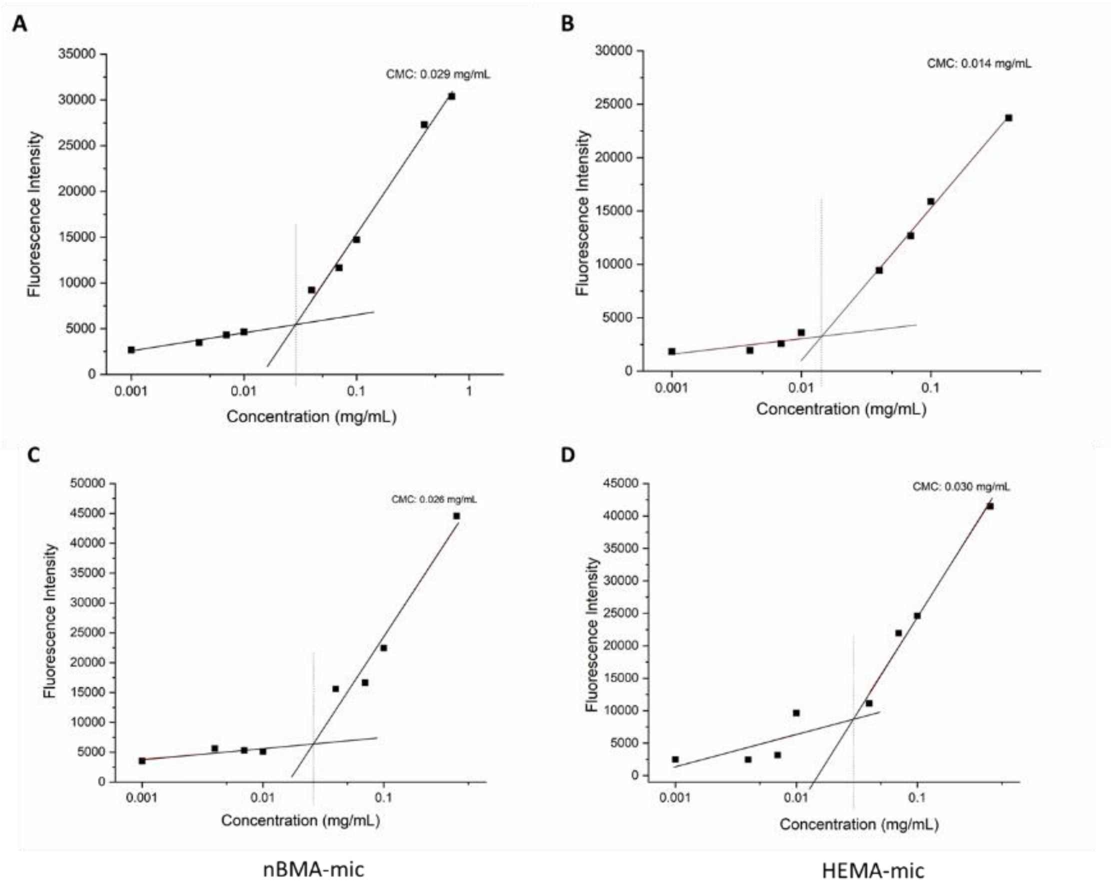

Figure S18. CMC determination.

Maximum of each fluorescence emission spectra was plotted versus the micelle concentration for each sample respectively at 25 °C with a Tecan Infinite M200 PRO microplate reader, using Nile Red as the fluorescence dye as a probe. A) LAMA-mic II, B) LAMA-mic I, C) *n*BMA-mic, D) HEMA-mic.

REFERENCES

1. McRae Page S, Martorella M, Parelkar S, Kosif I, Emrick T: **Disulfide cross-linked phosphorylcholine micelles for triggered release of camptothecin.** *Molecular pharmaceutics* 2013, **10:**2684-2692.
2. Larnaudie S: **Supramolecular cyclic peptide-polymer nanotubes as drug delivery vectors.** University of Warwick, 2017.
3. Bauer M, Lautenschlaeger C, Kempe K, Tauhardt L, Schubert US, Fischer D: **Poly(2-ethyl-2-oxazoline) as Alternative for the Stealth Polymer Poly(ethylene glycol): Comparison of in vitro Cytotoxicity and Hemocompatibility.** *Macromol Biosci* 2012, **12:**986-998.
4. Richter F, Martin L, Leer K, Moek E, Hausig F, Brendel JC, Traeger A: **Tuning of endosomal escape and gene expression by functional groups, molecular weight and transfection medium: a structure-activity relationship study.** *J Mater Chem B* 2020, **8:**5026-5041.
